# Combining Modules for Versatile and Optimal Labeling of Lactic Acid Bacteria: Two pMV158-Family Promiscuous Replicons, a Pneumococcal System for Constitutive or Inducible Gene Expression, and Two Fluorescent Proteins

**DOI:** 10.3389/fmicb.2019.01431

**Published:** 2019-06-26

**Authors:** Javier Nicolás Garay-Novillo, Diego García-Morena, José Ángel Ruiz-Masó, José Luis Barra, Gloria del Solar

**Affiliations:** ^1^Departamento de Biotecnología Microbiana y de Plantas, Centro de Investigaciones Biológicas, Consejo Superior de Investigaciones Científicas, Madrid, Spain; ^2^Centro de Investigaciones en Química Biológica de Córdoba (CIQUIBIC-CONICET), Córdoba, Argentina

**Keywords:** lactic acid bacteria, fluorescent labeling vectors, plasmid copy number, plasmid stability, plasmid fitness cost, mCherry and EGFP, fluorescent protein maturation, fluorescent protein stability

## Abstract

Labeling of bacterial cells with fluorescent proteins allows tracking the bacteria in competition and interactomic *in vivo* and *in vitro* studies. During the last years, a few plasmid vectors have been developed aimed at the fluorescent labeling of specific members of the lactic acid bacteria (LAB), a heterogeneous group that includes microorganisms used in the food industry, as probiotics, or as live vectors for mucosal vaccines. Successful and versatile labeling of a broad range of LAB not only requires a vector containing a promiscuous replicon and a widely recognized expression system for the constitutive or regulated expression of the fluorescence determinant, but also the knowledge of the main features of the entire plasmid/host/fluorescent protein ensemble. By using the LAB model species *Lactococcus lactis*, we have compared the utility properties of a set of labeling vectors constructed by combining a promiscuous replicon (pMV158 or pSH71) of the pMV158 plasmid family with the gene encoding either the EGFP or the mCherry fluorescent protein placed under control of promoter P_X_ or P_M_ from the pneumococcal *mal* gene cluster for maltosaccharide uptake and utilization, respectively. Some vectors carrying P_M_ also harbor the *malR* gene, whose product represses transcription from this promoter, thus enabling maltose-inducible synthesis of the fluorescent proteins. We have determined the plasmid copy number (PCN) and segregational stability of the different constructs, as well as the effect of these features on the fitness and fluorescence intensity of the lactococcal host. Constructs based on the pSH71 replicon had a high copy number (∼115) and were segregationally stable. The copy number of vectors based on the pMV158 replicon was lower (∼8–45) and varied substantially depending on the genetic context of the plasmid and on the bacterial growth conditions; as a consequence, inheritance of these vectors was less stable. Synthesis of the fluorescent proteins encoded by these plasmids did not significantly decrease the host fitness. By employing inducible expression vectors, the fluorescent proteins were shown to be very stable in this bacterium. Importantly, conditions for accurate quantification of the emitted fluorescence were established based on the maturation times of the fluorescent proteins.

## Introduction

Since the cloning and expression of the *gfp* cDNA encoding the green fluorescent protein (GFP) from the *Aequorea victoria* jellyfish in *Escherichia coli* (Prasher et al., 1992; Chalfie et al., 1994), the number of different applications of a battery of cnidarian-derived GFP-like fluorescent proteins (FPs) and their variants, as well as the range of bacterial species in which they can be used, have greatly increased. Among other applications, these FPs have been used in biosensors systems to monitor intracellular physiological parameters, like the pH, the oxygen level or the antioxidant activity (Potzkei et al., 2012; Crone et al., 2013; Germond et al., 2016). They are also useful as reporters of gene expression and transcription regulation (Chalfie et al., 1994; Webb et al., 1995; Ruiz-Cruz et al., 2010; Uliczka et al., 2011; Mohedano et al., 2014), or as biomarkers for imaging the subcellular location of fusion proteins and their targets (Webb et al., 1995; Phillips, 2001; Belardinelli and Jackson, 2017; Kapanidis et al., 2018), for direct detection and quantification of plasmid conjugation and plasmid loss (Nancharaiah et al., 2003; Sørensen et al., 2003; Bahl et al., 2004; Karimi et al., 2016), and for tracking the bacterial cells in *in vivo* and *in vitro* competition and interactomic studies (Singer et al., 2010; Campbell-Valois and Sansonetti, 2014; Jarchum, 2015; Russo et al., 2015). Imaging using these FPs as biomarkers is especially useful in living cells because is a non-destructive and minimally invasive method that does not require exogenously added substrates or cofactors (Chalfie et al., 1994).

GFP-like FPs share a similar 11-stranded β-barrel structure that encircles a central α-helix containing the chromophore (Zimmer, 2002; Miyawaki et al., 2012; Thorn, 2017). In order to become fluorescent, the newly synthesized FP polypeptide has to undergo a maturation process that, in addition to the folding into the β-barrel, involves formation of the chromophore by an autocatalytic mechanism that only requires molecular oxygen and consists of successive cyclisation, oxidation (usually the rate-limiting step) and dehydration reactions within a tripeptide located in the central α-helix (Zimmer, 2002; Miyawaki et al., 2012). To increase the spectrum of potential applications of the FP, new variants of the original proteins have been engineered that show alterations in their oligomeric state or in their intracellular stability, that produce different emission colors, that have increased brightness, photostability or maturation rates, that show improved excitation/emission patterns or protein folding, etc. Broad-host-range plasmids encoding a wide variety of FPs were constructed for their use in Gram-negative bacteria, where these proteins have been extensively characterized in order to find the optimal conditions for their *in vivo* and *in vitro* applications (Singer et al., 2010; Barbier and Damron, 2016). Moreover, a detailed study of the maturation kinetics of many of these FPs in living *E. coli* cells has recently been reported (Balleza et al., 2018). Red-shifted excitation green fluorescent variants of the wild-type (wt) GFP have been obtained that have Ser_65_ substituted by Ala, Gly, Ile, Thr, or Cys (Cormack et al., 1996). Among them, GFPmut1 (also termed enhanced GFP, EGFP), which contains both the S65T and F64L mutations resulting in a red-shifted excitation GFP with increased brightness and protein solubility, was early constructed and has been widely used (Miller and Lindow, 1997). Due to their spectral properties, these GFP variants can be properly combined with red FPs, like DsRed from Indo-Pacific reef coral *Discosoma* sp. and its improved monomeric variant mCherry (Shaner et al., 2004; Singer et al., 2010), for simultaneous dual fluorescent tagging of bacterial cells (Nancharaiah et al., 2003; Doherty et al., 2010).

Early, GFP (wt or mutant variants) was used both for subcellular protein localization and for reporting gene expression in *Bacillus subtilis* (Webb et al., 1995; Lewis and Errington, 1996), and GFP variants and mCherry have been tested for optimal or sporulation-stage-specific fluorescent labeling of this Gram-positive bacterium (Doherty et al., 2010; Overkamp et al., 2013). Also, two superfolder GFPs were constructed with specific codon adaptations for *B. subtilis*, which performed best in *Streptococcus pneumoniae* and in *Lactococcus lactis*, or for pneumococcus, which, however, performed best in *B. subtilis* (Overkamp et al., 2013). During the last two decades, there has been a growing use of GFP-like FPs (mainly some red-shifted excitation GFP variants and the red fluorescent mCherry protein) for cell tracking and for monitoring gene expression in Gram-positive bacteria belonging to the *Bifidobacterium* genus or to the heterogeneous group of LAB, which are commonly employed in the fermentation food industry or as probiotics (Landete et al., 2016, and references therein). A main drawback of using GFP-like FPs in bifidobacteria and LAB, which are generally cultivated under low-aeration or anaerobic conditions and produce lactic acid, is that the formation of the fluorophore depends on the presence of oxygen and its activity is sensitive to low pH (Pérez-Arellano and Pérez-Martínez, 2003; Doherty et al., 2010; Shinoda et al., 2018), so that neutralization and/or aeration steps subsequent to bacterial growth may be required for detecting and quantifying the fluorescence emission (Fernández de Palencia et al., 2000). Hansen et al. (2001) have reported, however, that maturation of the GFPmut3 chromophore in exponentially growing cells of *Streptococcus gordonii* occurs at levels of dissolved oxygen as low as 0.1 p.p.m. (achieved in conventionally prepared anaerobic media lacking reducing agents), whereas fluorescence is only prevented by adding L-cysteine to those media (i.e., at 0.025 p.p.m. oxygen). With respect to the effect of the acidification of the culture medium, these authors showed that inactivation of the GFPmut3 fluorophore by protonation could be rapidly reversed after a shift to neutral pH, provided that the *S. gordonii* cells had remained at low pH (<5) for up to 13 h, whereas fluorescence was not recovered for cells that had been maintained at low pH for 23 h (Hansen et al., 2001). Apart from those, very few studies have been performed on the features of different GFP-like FPs in LAB and there is a general lack of information relative to the maturation rate or to the intracellular stability of the FPs in these bacteria. In fact, these important characteristics, which can vary depending upon the external conditions and the genetic background, have generally been disregarded.

**FIGURE 1:**
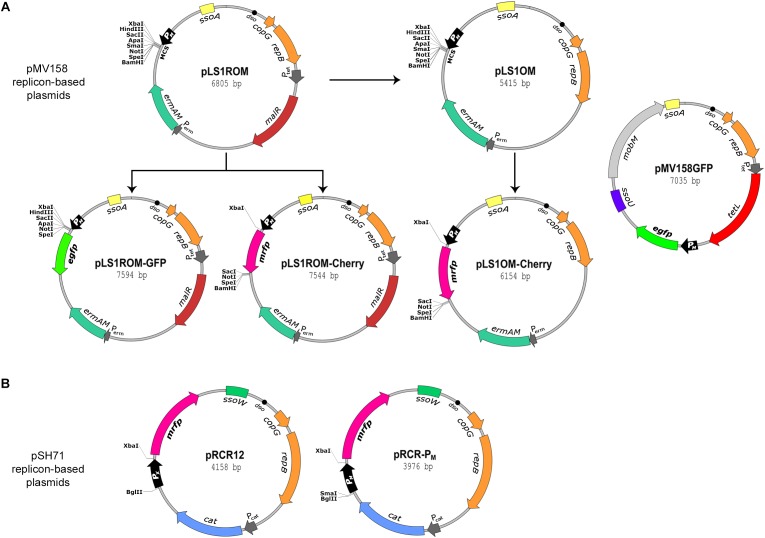
Maps of the plasmids used in this work. The plasmid vectors used in this work were grouped into those based on the pMV158 replicon **(A)** and those based on the pSH71 replicon **(B)**. Both replicons belong to the same pMV158 family of RC plasmids. The essential components of the plasmid replicons are referred as *dso*, double strand origin of replication, and as *copG* and *repB* genes, which are involved in copy number control and replication, respectively. *sso* refers to single strand origin of replication of types A, U, and W. *ermAM*, *cat*, and *tetL*, refers to Ery, Cm, and Tc resistant markers, respectively. Promoter sequences P_M_, P_X_, P_tet_, P_cat_, and P_*erm*_ are indicated by open arrows pointing in the direction of transcription. *malR* refers to the pneumococcal chromosomal gene encoding the MalR protein that, in the absence of maltose, represses transcription from P_M_ and P_X_. *mobM* refers to the plasmid conjugative mobilization gene of pMV158. EGFP- and mCherry-encoding genes are indicated as *egfp* and *mrfp*, respectively. Restriction sites of the multicloning sequence (MCS) of pLS1ROM and pLS1OM vectors are indicated. Unique restriction sites in pLS1ROM-GFP, pLS1ROM-Cherry, and in pLS1OM-Cherry remaining after insertion of *egfp* or *mrfp* gene casettes in the corresponding MCS are also indicated. Maps of pRCR12 and pRCR-P_M_ vectors include unique restriction sites of the MCS flanking the sequence of promoters P_X_ or P_M_, respectively.

Two main groups of plasmid constructs have been designed and used for fluorescent labeling of LAB, namely those derived from shuttle vectors and those containing a replicon functional in a wide range of these bacteria. Shuttle vector constructs contain a replicon functional in *E. coli* (usually p15A) along with a replicon effective in specific LAB (García-Cayuela et al., 2012; Campelo et al., 2014). Plasmid constructs that can be used for fluorescent labeling of a variety of LAB harbor theta-type (pAMβ1) or rolling circle (RC)-type (pMV158 family) promiscuous replicons (Fernández de Palencia et al., 2000; Pérez-Arellano and Pérez-Martínez, 2003; Ruiz-Masó et al., 2012; Campelo et al., 2014; Mohedano et al., 2014; Karimi et al., 2016). It is worth noting that the two pMV158-family RC replicons (pMV158 and pSH71) most often employed for the construction of promiscuous fluorescent labeling vectors (Figure 1) are functional not only in virtually all LAB tested but also in *E. coli* (Mohedano et al., 2014; Ruiz-Masó et al., 2015), which may facilitate the extraction and manipulation of the plasmidic DNA. Despite the growing number of recent studies that have made use of these tools for the fluorescent labeling of bacterial cells, only a few reports have dealt with the characterization of the plasmid constructs in the host LAB (Simon and Chopin, 1988; Ruiz-Masó et al., 2012; Campelo et al., 2014; Karimi et al., 2016), and information on the vector copy number and stability in the different target bacteria is actually missing.

Due to their recognition in a broad variety of hosts, promoters P_X_ or P_M_ of the pneumococcal *mal* gene cluster have frequently been used to direct expression of the genes encoding the FPs both in shuttle (García-Cayuela et al., 2012; Campelo et al., 2014) and in promiscuous (Fernández de Palencia et al., 2000; Nieto and Espinosa, 2003; Ruiz-Masó et al., 2012; Russo et al., 2015; Figure 1) vectors designed for the fluorescent labeling of LAB. In the chromosome of *S. pneumoniae*, P_X_ and P_M_ are divergent promoters that direct transcription of the operons involved in maltosaccharide uptake (*malXCD*) and utilization (*malMP*), respectively (Stassi et al., 1982; Nieto et al., 2001; Afzal et al., 2015). These two promoters are negatively regulated by the product of the *malR* gene of the *malAR* operon, which is also included into the *mal* gene cluster (Afzal et al., 2015). Protein MalR belongs to the LacI-GalR family of transcriptional regulators and acts as a repressor (in the absence of maltose or maltosaccharides) of the *malXCD* and *malMP* operons (Nieto et al., 2001), in contrast to MalR from *L. lactis*, which also belongs to the same family but behaves as an activator of genes involved in maltose transport when maltose is present (Andersson and Radström, 2002). Purified pneumococcal MalR binds with higher affinity to the *malMP* operator sequence (O_M_) than to the *malXCD* operator (O_X_) and, accordingly, represses P_M_ more strongly than P_X_ (Nieto et al., 2001).

In this work, we have used the *L. lactis* MG1363 host to compare the plasmid copy number (PCN), stability and fitness cost, as well as the level of production and the maturation rate and half-life of the FPs encoded by different labeling vectors (either previously reported or constructed in this work) that have been made by combining the indicated variants of the following modules: (i) a promiscuous replicon (pMV158 or pSH71) of the pMV158 family; (ii) a pneumococcal promoter (P_X_ or P_M_) directing transcription of the FP gene; (iii) the gene encoding a FP (EGFP or mCherry); (iv) the pneumococcal gene encoding the MalR transcriptional repressor, which can be either absent or present in constructs harboring promoter P_M_, thus making fluorescent labeling unrepressed or maltose-inducible, respectively, and (v) an antibiotic (chloramphenicol, erythromycin, or tetracycline) resistance selectable marker. We also show the utility of the labeling vector carrying the maltose-inducible *egfp* expression system for direct and easy quantification of the plasmid loss.

## Materials and Methods

### Bacterial Strains, Growth Conditions, and Plasmids

*L*. *lactis* subsp. *cremoris*, strain MG1363 (Gasson, 1983) was cultivated in M17 medium supplemented with 4% maltose (M17M) for optimal induction conditions (Viegas et al., 2004), or 4% glucose (M17G), unless otherwise indicated, at 30°C, without aeration. *S. pneumoniae* 708 (Lacks and Greenberg, 1977) was cultivated in AGCH broth (Lacks et al., 1986) supplemented with 0.3% sucrose and 0.2% yeast extract (AGCH_SY_ hereafter) at 37°C without shaking. When needed, chloramphenicol (Cm), erythromycin (Ery), and tetracycline (Tc) were added at the indicated concentrations. Growth was monitored with the aid of Spectronic 20D+ equipment by checking optical density (OD) at 660 nm for *L. lactis* and at 650 nm for *S. pneumoniae*.

Absorbance and fluorescence of the samples were measured in a Varioskan Flash system (Thermo Fisher Scientific, Waltham, MA, United States). This system provides quantitative data of cell density by measuring OD at the appropriate wavelength (λ), mCherry fluorescence (excitation λ, λ_ex_, of 587 nm, and emission λ, λ_em_, of 612 nm), and EGFP fluorescence (λ_ex_ = 488 nm and λ_*em*_ = 515 nm).

Plasmid constructions used throughout this study, as well as their relevant features, are listed in Table 1.

**Table 1:** Plasmids used in this study.

Plasmids				
Plasmids	Description	Characteristics	References	Functionality tested in
pMV158 (5541 pb)	Natural plasmid isolated from *Streptoccocus agalactiae*; Tc^R^		Burdett, 1980	A variety of species of the Firmicutes, Actinobacteria and Proteobacteria phyla
pLS1 (4408 bp)	Non-mobilizable pMV158 derivative plasmid (Δ*mobM*, Δ*ssoU*); Tc^R^		Stassi et al., 1981	A variety of species of the Firmicutes, Actinobacteria and Proteobacteria phyla
pLS1ROM (6805 bp)	Ery^R^, *malR*, P_M_ -MCS, pMV158 replicon	Gene cloning and maltose-inducible expression from promoter P_M_ in Firmicutes	Ruiz-Masó et al., 2012	*S. pneumonia, L. lactis, Enterococcus faecalis, Lactobacillus casei*, *B. subtilis.*
pLS1OM (5415 bp)	Ery^R^, P_M_ -MCS, pMV158 replicon	Gene cloning and constitutive expression from promoter P_M_ in Firmicutes lacking the pneumococcal *malR* gene	This work	*L. lactis, S. pneumoniae*
pLS1ROM-GFP (7594 bp)	Ery^R^, *malR*, P_M_-MCS-*egfp*, pMV158 replicon	Maltose-inducible *egfp* expression from P_M_ in Firmicutes	Ruiz-Masó et al., 2012	*S. pneumoniae, L. lactis, E. faecalis*, *Lb. casei*, *B. subtilis.*
pLS1ROM-Cherry (7544 bp)	Ery^R^, *malR*, P_M_-MCS-*mrfp*, pMV158 replicon	Maltose-inducible *mrfp* expression from P_M_ in Firmicutes	This work	*L. lactis, S. pneumoniae.*
pLS1OM-Cherry (6154 bp)	Ery^R^, P_M_-MCS-*mrfp*, pMV158 replicon	Constitutive expression of *mrfp* from P_M_ in Firmicutes lacking the pneumococcal *malR* gene	This work	*L. lactis, S. pneumoniae.*
pMV158GFP (7035 bp)	Tc^R^, P_M_-*egfp*, *mobM*, pMV158 replicon	Mobilizable plasmid expressing *egfp* constitutively from P_M_ promoter in Firmicutes lacking the pneumococcal *malR* gene	Nieto and Espinosa, 2003	Firmicutes (*S. pneumoniae*, *L. lactis, E. faecalis), Escherichia coli.*
pRCR (3960 bp)	Cm^R^, pSH71 replicon	Promoter-probe vector carrying a promoterless *mrfp* gene	Mohedano et al., 2014	*E. coli, L. lactis, Leuconostoc lactis, Lactobacillus species, Pediococcus parvulus*.
pRCR12 (4158 bp)	Cm^R^, P_X_-*mrfp*, pSH71 replicon	Constitutive *mrfp* expression from P_X_ in Firmicutes lacking the pneumococcal *malR* gene	Russo et al., 2015	*E. coli, L. lactis, Lactobacillus species, S. pneumoniae, P. parvulus, B. subtilis*
pRCR-P_M_ (3976 bp)	Cm^R^, P_M_-*mrfp*, pSH71 replicon	Constitutive *mrfp* expression from P_M_ in Firmicutes lacking the pneumococcal *malR* gene	This work	*L. lactis, S. pneumoniae*

### Plasmid and Genomic DNA Preparation

*L. lactis* plasmid DNA was extracted using Midiprep kit (Genomed) optimized for this bacterium. Cells resuspended in the solution provided by the kit were incubated with 50 mg/mL lysozyme at 37°C for 30 min before proceeding with the lysis step. Genomic DNA (gDNA) from *L. lactis*, used as template for real-time quantitative PCR (qPCR), was isolated by using the Wizard Genomic DNA Purification kit (Promega). Prior to the lysis step, collected cells were resuspended in a solution containing EDTA 50 mM and lysozyme 30 mg/mL, and incubated at 37°C for 40 min.

Purified gDNA was digested with EcoRI, which linearizes the plasmid DNAs analyzed in this study but leaves intact the plasmidic and chromosomal amplicons (i.e., the template DNA segments to be amplified in the qPCR assays). This method was developed to obtain accurate qPCR-based copy number results for plasmids (Providenti et al., 2006). Concentration of the gDNA was determined with a Qubit fluorometer by using the Qubit HS dsDNA Assay kit (Molecular Probes).

*S. pneumoniae* plasmid DNA was extracted with the High Pure Plasmid Isolation kit (Roche) with the modifications described in Ruiz-Cruz et al. (2010).

In all cases, DNA integrity was checked by 0.8% agarose gel electrophoresis and DNA staining with GelRed (Biotium).

### Plasmid Constructions

To construct the pLS1OM vector, an inverse PCR approach (Ochman et al., 1988) was used by employing phosphorylated divergent primers DelmalR1 and DelmalR2 (Supplementary Table S1), and DNA from pLS1ROM (Ruiz-Masó et al., 2012) as template. The amplification reaction with the Phusion polymerase (Thermo Fisher Scientific) yielded a linear DNA fragment containing the pLS1ROM sequence except the P_tet_ promoter and the downstream *malR* gene. The amplified fragment was gel-purified and subjected to auto-ligation to render circular plasmids molecules that were used to transform *S. pneumoniae* 708. Final plasmid vector pLS1OM is 5.4 kb in size.

To construct pLS1ROM-Cherry, pLS1OM-Cherry, and pRCR-P_M_ vectors, DNAs from pLS1ROM, pLS1OM, and pRCR (Mohedano et al., 2014) were digested with XbaI, which generates 5′-P protruding ends, and with SmaI, which leaves 5′-P blunt ends. The *mrfp* gene, encoding the mCherry protein, was PCR-amplified by using specific primers Fmcheclon and 5′-phosphorylated Rmcheclon (Supplementary Table S1), and DNA from pRCR12 as template. The P_M_ promoter region was PCR-amplified by using specific primers SecMCSGFP and 5′-phosphorylated M2 (Supplementary Table S1), and DNA from pLS1ROM as template. The resultant fragments (∼0.9 kb for *mrfp* and ∼0.4 kb for P_M_) were gel-purified and subsequently digested with XbaI, which yielded a protruding 5′-P end, and ligated to the pLS1ROM, pLS1OM, or pRCR larger XbaI-SmaI fragments. The ligation mixture was used to transform *S. pneumoniae* 708 competent cells. The presence of phosphorylated 5′-ends allows T4 DNA ligase to seal all nicks in the recombinant DNA. In fact, the presence of nicks at fixed sites decreases the DNA ability to transform *S. pneumoniae*, due to the particular mechanism of entry and reconstitution of plasmidic DNA monomers by natural transformation of this bacterium (López et al., 1982; Ruiz-Masó et al., 2017). The correct nucleotide sequence of all new constructs was confirmed by automated DNA sequencing.

All plasmid constructions were obtained in *S. pneumoniae* and then introduced by electrotransformation in *L. lactis*.

### Transformation of Bacterial Species With Plasmid DNA

*L. lactis* electrocompetent cells were obtained following the protocol described in de Felipe et al. (1995) and were used immediately after preparation. Electrotransformation conditions were as follows: 200 Ω, 25 μF and 12.5 kV/cm, obtaining a time constant of ∼4.5–5 ms. Transformant cells were recovered in 1.5 mL of M17 plus 0.5% glucose and 1% sucrose, and incubated 2 h at 30°C without aeration. For cells transformed with pRCR or derivatives, expression of the Cm^R^ *cat* gene was induced with Cm 0.5 μg/mL within the last 30 min of incubation. Transformants were selected using M17 0.5% glucose agar plates containing Ery 5 μg/mL for pLS1ROM and derivatives, Cm 5 μg/mL for pRCR and derivatives, and Tc 1 μg/mL for pMV158, pLS1 and pMV158GFP. Plasmid content of the colonies was analyzed by the Miniprep kit (Roche) optimized for *L. lactis*, and the integrity of the DNA was checked by 0.8% agarose gel electrophoresis.

Competent cells of *S. pneumoniae* 708 were prepared and transformed as described (López et al., 1982). Transformants were selected with 3 μg/mL Cm or 1 μg/mL Ery. Cells carrying the desired constructions were detected by colony PCR using Taq DNA polymerase (Roche), and the correct insert’s nucleotide sequence was confirmed by automated DNA sequencing.

### Estimation of the Growth Rate Constant of *L. lactis*

The bacterial growth rate constant (*μ*) was calculated during the exponential growth phase. Lactococcal strains without plasmid or carrying the different constructs were grown in M17G or M17M supplemented (when required) with the corresponding antibiotic for plasmid selection, until an OD of 0.4 was reached. After collecting and washing the cells with PBS, cultures were diluted 20-fold in fresh M17G or M17M either containing or not the corresponding antibiotic. Next, 200 μL triplicates of each culture were transferred to a 96-well optical bottom plate and incubated at 30°C for 800 min in the Varioskan (see section Bacterial Strains, Growth Conditions, and Plasmids). Cell growth was monitored by measuring the OD_660_ every 20 min. The *μ* value of exponentially growing *L. lactis* cultures was determined from the following equation:

(1) $$\ln({\mathrm{OD}}_{t}/{\mathrm{OD}}_{0})=\mu \;(t-t_{0})$$

where *OD*_0_ and *OD_t_* correspond to the optical density of the cultures at the times *t*_0_ and *t*, respectively, and *μ* is the slope of the linear regression fit in the plot of the experimental values of *ln*(*OD_t_*/*OD*_0_) against the incubation time (*t*).

Analysis of the growth curves in M17M showed the existence of two distinct exponential phases. The first phase, with a higher apparent *μ*, coincides with the use of the trace amounts of glucose and trehalose that we have found to be present in the M17M medium. The subsequent phase of slower growth corresponds to the utilization of maltose by the bacterial cells. The presence of traces of sugars in M17M, and their preferred consume with respect to maltose, was detected through the analysis of culture supernatants by gas chromatography-mass spectrometry (not shown). Therefore, for calculating *μ* in the presence of maltose as carbon source we only considered cell growth measured after depletion of the residual glucose and trehalose, from an OD ∼0.3 to the end of the exponential phase.

### Determination of Plasmid Copy Number in *L. lactis* Cells by qPCR and Agarose Gel Quantification

For the determination of the relative PCN by qPCR, different primer pair sets specific to the PcrA helicase single-copy reference gene (*pcrA*) of *L. lactis* MG1363 (Wegmann et al., 2007), to the replication protein RepB gene (*repB*) of pMV158, and to the mCherry–encoding gene (*mrfp*) were designed. A previously designed primer pair specific to the Tc resistance (Tc^R^) TetL protein gene (*tetL*) of pMV158 was also used (Ruiz-Masó et al., 2017). Oligonucleotide primers are listed in Supplementary Table S1. Criteria used during primer design were that primers had predicted Tm of ∼59°C and that they generated amplicons ∼140 bp in length.

qPCRs were conducted as described (Ruiz-Masó et al., 2017). Decimally diluted EcoRI (Nzytech)-digested gDNA preparations (17, 1.7, and 0.17 ng per reaction) were analyzed using 0.5 μM of the specific forward and reverse primers of either primer pair used. Three independent qPCR trials were conducted for each template source. In each trial, triplicate samples of the three different amounts of template were analyzed. Control samples without template DNA were also analyzed.

PCN of the different constructions was calculated using equation:

(2) $$\mathrm{PCN}={\left(1+E_{\mathrm{pcrA}}\right)}^{{\mathrm{Ct}}_{\mathrm{pcrA}}}/{\left(1+E_{\mathrm{plasmid}}\right)}^{{\mathrm{Ct}}_{\mathrm{plasmid}}}$$

where *E*_pcrA_ and *E*_plasmid_ are, respectively, the PCR amplification efficiencies of the chromosomal and plasmid amplicons, and *Ct_pcrA_* and *Ct*_plasmid_ are the mean threshold cycle values obtained for the corresponding amplicons. An average PCN value was calculated for each of the three template concentrations analyzed. In addition, the mean and standard deviation of the three independent experiments (biological replicates) was calculated for each PCN determination. *E* values of target (*E*_plasmid_) and reference (*E*_pcrA_) sequences were empirically calculated for each qPCR trial. For that purpose, mean Ct values were plotted against the logarithm of the amount of total DNA template in the assay. From the slope of the curve generated by linear regression of the plotted points, the PCR amplification efficiency was determined according to the equation:

(3) $$E=10^{-1/\mathrm{slope}}-1$$

Although the *E*-values for both amplicons were higher than 0.9, we have chosen a mean E value of all experiments to calculate the relative PCN as it allows taking into account the slight differences between *E*_target_ and *E*_reference_ that we have observed.

For PCN determination by agarose gel quantification, gDNAs, extracted as depicted in section Plasmid and Genomic DNA Preparation, were loaded into 0.8% agarose gels, run for 60 min at 6.5 V/cm and dyed with GelRed for 10 min. Gels were visualized with the aid of a Gel Doc (Bio-Rad). Non-saturated images of the gels were analyzed with the Quantity One^®^ v4.5.2 software (Bio-Rad). Plasmidic and chromosomic DNA bands were identified and analyzed following a Gaussian distribution model for determination of the different lane densities. For PCN determination, plasmid to chromosomal DNA ratios, normalized to correct for plasmid size differences, were quantitatively compared with the same ratio of plasmid pRCR12, whose PCN in *L. lactis* determined by qPCR was considered as a reference. PCN of pRCR12 was taken as a reference value due to the precision of its determination, with a low standard deviation that represents less than 10% of the mean. Only the plasmid band corresponding to the covalently-closed circles was considered for the comparative quantification.

### Determination of Plasmid Structural and Segregational Stability in *L. lactis*

Plasmid structural and segregational stability was determined in exponentially growing cultures of *L. lactis* containing different plasmids. Lactococcal cells were grown up to an OD of 0.4 in M17G in the presence of selective pressure for the antibiotic resistant marker carried by the resident plasmid. Next, the strains were sub-cultured for ten generations by 1:1000 dilution in medium without antibiotic and allowed to reach again an OD of 0.4. The process was repeated for at least 100 generations of serial cultivation. Several aliquots of liquid culture were taken every ten generations in order to determine the structural and segregational stability of the plasmids.

The plasmid structural stability was determined by analyzing the plasmid integrity in bacterial gDNA preparations. Aliquots of 2 mL of the cultures at an OD = 0.4 were used for gDNA preparation as depicted in section Plasmid and Genomic DNA Preparation. The gDNA samples were subjected to electrophoresis on 0.8% agarose gels, stained with GelRed, and visualized with the aid of a Gel Doc system. The segregational stability of the plasmids was analyzed by three different methods: (i) determination of the percentage of colony forming units (cfu) that express the antibiotic resistant marker carried by the plasmid; (ii) quantification of the specific fluorescence (ratio between the fluorescence intensity and the OD) emitted by the cultures; and iii) determination of the percentage of fluorescent cells by fluorescence microscopy.

In the case of the *L. lactis* strains carrying pLS1ROM, pMV158 or pLS1, cells were grown in M17G in the absence of selective pressure for 150 generations, and samples were taken only to analyze the fraction of antibiotic-resistant cfu. The *L. lactis* strains harboring pMV158GFP, pRCR12, or pLS1ROM-GFP were also grown for 150 generations in the absence of selective pressure and analyzed for determination of the percentage of antibiotic-resistant cfu and for quantification of the fluorescence emission. Triplicate 200 μL aliquots were collected, and the cells were washed, resuspended in PBS and loaded in a 96-well plate, which was read for absorbance (480 nm) and fluorescence in the Varioskan microplate reader. In the case of cells containing pLS1ROM-GFP, the expression of *egfp* from promoter P_M_ in the culture aliquots was induced with 4% maltose as indicated below, before analyzing the bacterial fluorescence using either the Varioskan or the fluorescence microscope.

To determine the percentage of fluorescent cells in the strains harboring pMV158GFP, pRCR12 and pLS1ROM-GFP, 10-fold concentrated cell samples in PBS were visualized with the aid of an optical fluorescence microscope (Leica DM1000) equipped with a camera with a CCD sensor. After analyzing the fluorescence microscopy images of pLS1ROM-GFP-containing *L. lactis* cells grown in M17G, only a negligible fraction (∼1%) of cells yielded EGFP fluorescence (potentially due to leaky expression of the *egfp*), at least under the image acquisition conditions employed. Moreover, after induction of *egfp* expression for 45 min in the presence of 4% maltose, as low as ∼8% of the *L. lactis*/pLS1ROM-GFP cells failed to produce EGFP fluorescence levels above the detection threshold. Similarly, we observed ∼5% of non-fluorescent cells in cultures in M17G of *L. lactis* carrying the stable pMV158GFP plasmid that determines expression of *egfp* irrespective of the presence of maltose. Hence, only a small fraction of the cells expressing *egfp* contains levels of mature EGFP below the detection threshold (false negatives).

In all cases, we determined the fraction of plasmid-containing cells after growing for *n* generations in the absence of selective pressure (*P_n_*). The experimental plasmid loss rate per cell and generation (*L_*ex*_*) was calculated from the equation (Gerdes et al., 1985; del Solar et al., 1993):

(4) $$P_{n}=P_{0}{\left(1-L_{\mathrm{ex}}\right)}^{n}$$

where *P*_0_ is the initial fraction of plasmid-containing cells. The previous formula can be converted into a linear function by taking logarithms,

(5) log (Pn/P0)=n log(1−Lex)

where *log* (1 – *L_*ex*_*) is the slope of the linear regression fit in the plot of the experimental values of *log* (*P_n_*/*P*_0_) against *n*.

Theoretical plasmid loss rates (*L_*th*_*) due to random distribution of the plasmid copies between the daughter cells is given by the equation (Nordström and Austin, 1989):

(6) $$L_{\mathrm{th}}={\left(1/2\right)}^{2\mathrm{PCN}}$$

### Induction of FP Genes Under Control of Promoter P_M_ in *L. lactis*

Inducible expression of FP genes *egfp* and *mrfp* was assayed in the lactococcal strains carrying pLS1ROM-GFP or pLS1ROM-Cherry. Bacterial cells were grown in the appropriate selective medium (see section Bacterial Strains, Growth Conditions, and Plasmids) to an OD of 0.4. Then, they were washed once with PBS and diluted to reach an OD of 0.2 in the appropriate broth supplemented with antibiotic and with 4% glucose or 4% maltose (inducer). After 4 h, 200 μL triplicates of each culture were harvested, and the cells washed, resuspended in the same volume of PBS and transferred to a 96-well optical bottom plate. The bacterial growth (480 nm) and the levels of the EGFP or mCherry were tested with the Varioskan (see section Bacterial Strains, Growth Conditions, and Plasmids).

### Estimation of the FP Maturation Times by Translational Arrest With Erythromycin

*L. lactis*/pRCR-P_M_ or *L. lactis*/pMV158GFP strains were grown in AGCH medium supplemented with 0.5% glucose, 0.2% of yeast extract (AGCH_GY_ hereafter), and the corresponding antibiotic. This medium was chosen because it provides a background fluorescence much lower than M17, and is hence more suitable for real-time fluorescence measurements. Cells were grown at 30°C to an OD_650_ of 0.85, thus allowing for maximum fluorescence level while avoiding extensive acidification of the culture medium, the pH of which was maintained near the neutrality. Next, 5 μg/mL Ery was added to the cultures to initiate the translational arrest for investigating EGFP and mCherry maturation. Immediately after addition of Ery, six 200 μL replicates of the cultures were loaded in a 96-well optical bottom plate and incubated at 30°C in the Varioskan. Cell density was monitored by measuring absorbance at 650 nm. EGFP and mCherry fluorescence emitted, respectively, by the lactococcal cells carrying pMV158GFP or pRCR-P_M_ was measured at different time intervals over an 8-h period. After the time interval of analysis, no significant variations of the cell density were observed. Viable cells were counted by spreading on selective agar plates 100 μL aliquots of suitable dilutions of culture samples taken at 60 min intervals. More than 90% of cells of both strains remained viable after 8 h of incubation at the indicated conditions.

Prior to data analysis, initial EGFP and mCherry fluorescence values, measured immediately after addition of Ery, were subtracted. Then, the corrected fluorescence data were fitted by using the exponential function:

(7) $$F_{t}=1-e^{\left(-t/\mathrm{MT}\right)}$$

where *F_t_* represents the fraction of the maximal fluorescence intensity at time *t*. The maturation time (*MT*) was determined as the characteristic parameter of the exponential fit, indicating the point in time at which about 63% of the maximum fluorescence intensity was reached (*t*_63_).

### Determination of the Half-Life of EGFP and mCherry in *L. lactis*

*L. lactis*/pLS1ROM-GFP and /pLS1ROM-Cherry strains were grown up to an OD_660_ of 0.4 in M17G supplemented with 5 μg/mL Ery. Then, the cells were washed with PBS and diluted to an OD of 0.1 in 30 mL of M17M plus 5 μg/mL Ery, in order to induce the production of FPs. The induction was prolonged for 8 h at 30°C to ensure a sufficient production of the FPs. After that, the cells were harvested, washed with PBS, and concentrated 10-fold in 3 mL of AGCH (Lacks et al., 1986) without the supplementation of any sugar or other components that could be used by bacterial cells as a carbon source. The shift to AGCH stops bacterial growth and induction of the *egfp* or *mrfp* expression. Consequently, the production of FPs was prevented over the period of time during which the fluorescence intensity decline was monitored. After the shift, 200 μL triplicates of the concentrated cell suspension were loaded in a 96-well optical bottom plate and incubated at 30°C in the Varioskan equipment. Cell density was monitored by measuring absorbance at 650 nm. EGFP or mCherry levels were monitored by measuring fluorescence at the appropriate excitation and emission wavelengths every 60 min over a 12 h period. To tests cell viability over this period, 2 μL aliquots of suitable dilutions of the concentrated cell suspension, taken immediately after the shift and at 60 min intervals, were added to 98 μL of PBS and spread on selective agar plates. Cell counting showed that more than 95% of cells of both strains remained viable after 12 h of incubation at the indicated conditions. After blank correction, specific fluorescence values relative to absorbance at 650 nm were determined.

The half-time (*t*_1/2_) of EGFP or mCherry inside lactoccocal cells, defined as the time it takes for the fluorescence emitted by EGFP or mCherry to be reduced by 50%, was estimated according to the equation:

(8) Ft=Fmax1/2t/t1/2

This equation can be converted into a linear function by taking logarithms:

(9) $$\log(F_{t}/F_{\max})=-\mathrm{tlog}2/t_{1/2}$$

The specific fluorescence values (*F_t_*) normalized to the maximum relative fluorescence (*F_max_*), represented as *log (F_t_/F_max_)*, were fitted by linear regression against time (*t*) during a certain interval initiated when the *F_max_* value was reached. In the case of the cultures expressing *egfp*, the *F_max_* value of the FP was reached shortly after the shift to AGCH. However, in the case of the cultures expressing *mrfp*, and due to the slower maturation rate of the mCherry FP, the *F_max_* value was reached 6 h after the shift to AGCH. In Equation (9), the value: –*log*2*/t*_1/2_ represents the slope (*m*) of the linear regression fit, so that the half-life time (*t*_1/2_) can be calculated from the expression:

(10) $$t_{1/2}=-\log2/m$$

### Statistical Analysis

Statistical analyses were carried out using SigmaPlot software (v12.5, Systat software, San Jose, CA, United States). The differences among PCNs as well as the differences in bacterial growth rate constant were analyzed by one-way ANOVA. The level of significance was set at *P* < 0.05.

## Results

### Construction of New Vectors Based on Either the pMV158 or the pSH71 Replicon

In order to increase the versatility of the promiscuous labeling vectors based on the pMV158-replicon family and to facilitate their characterization, we generated new constructions carrying either the pMV158 replicon (pLS1OM, pLS1ROM-Cherry, pLS1OM-Cherry; Figure 1A) or the pSH71 replicon (pRCR-P_M_; Figure 1B).

We first removed the *malR* gene from pLS1ROM to generate pLS1OM, a vector in which the P_M_ promoter becomes released from the tight transcriptional control exerted by the presence in *cis* of the repressor gene. In fact, pLS1OM is expected to yield unrepressed (hereafter referred to as constitutive) transcription from P_M_ irrespective of the presence of maltose, provided that the bacterial host lacks a similar MalR repressor recognizing O_M_.

Next, labeling vectors pLS1ROM-Cherry and pLS1OM-Cherry were constructed by cloning the *mrfp* gene encoding the mCherry FP under control of the P_M_ promoter of pLS1ROM or pLS1OM, respectively (Figure 1A). Hence, pLS1ROM-Cherry and pLS1OM-Cherry should enable, respectively, the maltose-inducible and constitutive red-fluorescent labeling of *L. lactis* and other LAB lacking a MalR repressor that binds to O_M_ when maltose is absent. It is worth noting that, in contrast, both these plasmids provide maltose-inducible expression of the *mrfp* gene in the pneumococcal host, due to the presence of *malR* in the chromosome of in this bacterium (Nieto et al., 2001; not shown).

New plasmids pLS1ROM-Cherry and pLS1OM-Cherry, together with previously constructed pMV158GFP (Nieto and Espinosa, 2003) and pLS1ROM-GFP (Ruiz-Masó et al., 2012), offer a battery of pMV158 replicon-based fluorescent labeling vectors (Figure 1A) differing among them in: (i) the antibiotic resistance marker they carry (namely, Tc^R^ in pMV158GFP, and Ery^R^ in pLS1ROM-GFP, pLS1ROM-Cherry and pLS1OM-Cherry); (ii) the FP encoded (EGFP in pMV158GFP and pLS1ROM-GFP, and mCherry in pLS1ROM-Cherry and pLS1OM-Cherry); (iii) the constitutive (pMV158GFP and pLS1OM-Cherry) or maltose-inducible (pLS1ROM-GFP and pLS1ROM-Cherry) expression of the FP gene in *L. lactis*; and (iv) the presence (pMV158GFP) or absence (pLS1ROM-GFP, pLS1ROM-Cherry, and pLS1OM-Cherry) of the pMV158 single-strand origin *U* (*ssoU*), which is widely recognized in Firmicutes as an efficient signal for the initiation of the lagging-strand synthesis during RC plasmid replication (Lorenzo-Díaz and Espinosa, 2009; Ruiz-Masó et al., 2015). Plasmids pLS1ROM-GFP, pLS1ROM-Cherry, and pLS1OM-Cherry only harbor the pMV158 *ssoA*, which is fully functional in streptococci and is also recognized, although less efficiently, in *L. lactis*, so that some single-stranded (ss) DNA corresponding to plasmid replication intermediates accumulates in the latter bacterium (Meijer et al., 1995).

Based on the pSH71 replicon, a new vector (pRCR-P_M_) for red-fluorescent labeling of LAB was constructed by cloning the P_M_ promoter region upstream from the *mrfp* gene of the pRCR promoter-probe plasmid (Figure 1B). Thus, pRCR-P_M_ and pRCR12 only differ in that transcription of *mrfp* is directed by P_M_ and P_X_, respectively. Both pRCR12 and pRCR-P_M_ harbor the pSH71-*ssoW* (Figure 1B), which is efficiently recognized in lactococci (but not in other LAB) as a lagging-strand origin (Seegers et al., 1995). The analysis of the fluorescence emission intensities of lactococcal cells carrying either pRCR12 or pRCR-P_M_ allows comparing the relative strength of promoters P_X_ and P_M_, and hence selecting the most suitable labeling vector for a particular application in this bacterium.

### Effect of the Presence of Vectors Carrying pMV158-Family Replicons on Host Fitness

**FIGURE 2:**
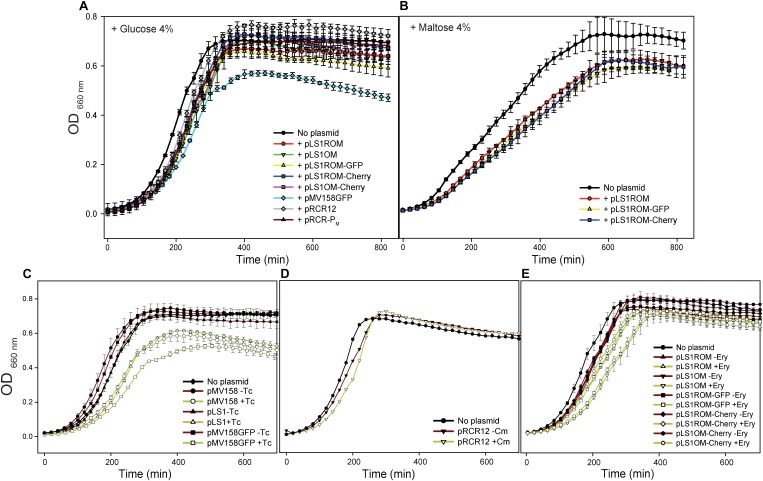
Effect of the presence of the plasmid vectors on the bacterial host fitness. Strains of *L. lactis* MG1363 lacking any plasmid or harboring the indicated plasmids were grown in M17 supplemented with glucose **(A)** or maltose **(B)**, and in the presence of selective pressure for the antibiotic resistant marker codified by the resident plasmid. In addition, *L. lactis* strains carrying pMV158, pLS1, pMV158GFP **(C)**, pRCR12 **(D)**, or a plasmid of the pLS1ROM/OM series **(E)** were grown in M17G in the presence (+) or in the absence (−) of selective pressure. Optical density at 660 nm was measured every 20 min using a Varioskan microplate reader. Plots display representative growth curves of each strain as obtained from one of at least three independent experiments (biological samples), with the symbols and vertical bars representing the average and errors of three technical triplicates.

**FIGURE 3:**
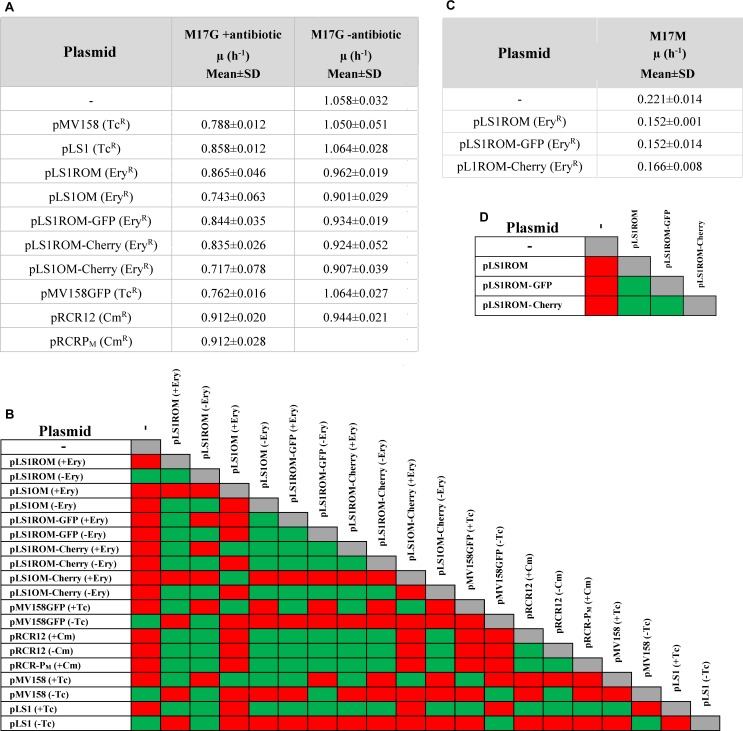
Analysis of the growth rate constant (μ) determined for any of the plasmid-containing strains. The μ values of *L. lactis* harboring different vectors in M17G **(A)** or M17M **(C)** are shown. In **(A)** the lactococcal strains were grown in the presence (+) or in the absence (−) of selective pressure for the antibiotic resistant marker codified by the resident plasmid. In **(C)** the plasmid-carrying strains were grown in the presence of the corresponding antibiotic. The values given in the tables of **(A,C)** are expressed as mean ± standard deviation (SD) of, at least, three independent experiments. A correlation matrix of the statistical significance of the growth rates of the lactococcal strains containing the studied plasmid vectors in M17G **(B)** or M17M **(D)** is displayed. Green, no statistically differences could be inferred (*P* > 0.05); red, statistically differences could be inferred (*P* < 0.05).

To evaluate the potential fitness cost associated with the plasmid carriage in the lactococcal host, we compared the growth curve of the plasmid-free strain with those of the strains harboring the different constructs depicted in Figure 1. To this end, the different bacterial strains were grown separately in M17G under selective pressure for the antibiotic resistance marker carried by the hosted plasmid (or in the absence of any antibiotic in the case of the plasmid-free strain), and the change in the OD of the bacterial cultures was monitored over time (Figure 2A). Addition of the antibiotics to the culture media ensured the presence of segregationally-unstable plasmids in the dividing bacterial cells, so that the determined growth rates were only contributed by the plasmid-containing cells, although a potential noxious effect of the antibiotic on the bacteria expressing the corresponding resistance marker gene could not be ruled out. At least three independent assays were carried out for each strain. The exponential-phase growth rate constant determined for any of the plasmid-containing strains was significantly lower than that of the plasmid-free strain (*P* < 0.05; Figure 3A,B), the slowest growth corresponding to the lactococcal cells that carried pMV158GFP, pLS1OM, or pLS1OM-Cherry.

To know whether the observed slowdown of the plasmid-carrying strains in selective media was due to the metabolic burden imposed by the vectors on the bacterial host or to the deleterious effect of the antibiotic itself, log-phase cultures (OD ∼0.4) of these strains were diluted 1:20 in media with or without antibiotics, and the bacterial growth was monitored for about five generations (Figure 2C–E). No significant differences between the growth rate constants in selective and non-selective media were found for the pRCR12 lactococcal host, which carries a Cm-inducible *cat* gene determining Cm^R^ (Figure 2D, 3A,B). These results indicate that: (i) expression of the plasmidic *cat* determinant does not burden significantly the host cell, and (ii) the Cm acetyltransferase encoded by the *cat* gene efficiently inactivates the antibiotic preventing its detrimental effect on the bacterial cell. In contrast, the growth rate constant of the pMV158GFP-carrying strain increased significantly when the antibiotic (Tc) was absent, becoming undistinguishable from that of the plasmid-free strain (Figure 2C, 3A,B). Removal of Tc also resulted in a similar increase in the growth rate of lactococcal strains that contained the pMV158 parental plasmid or its derivative pLS1 (Figure 2C, 3A,B), both harboring (like pMV158GFP) the constitutively-expressed *tetL* Tc^R^-determinant which encodes a Tc-efflux membrane protein (Lacks et al., 1986). Hence, the slowdown observed when these strains are grown in the presence of Tc seems to arise from the nocuous effect caused by the antibiotic before it can be expelled from the cell, rather than from the plasmid carriage itself. Finally, we also analyzed the effect of the presence of the antibiotic in the growth rate of the lactococal strains containing any of the pLS1ROM/OM series plasmids, which constitutively express the *ermAM* Ery^R^ marker. Although these plasmids showed a slightly unstable inheritance in the lactococcal host **(**see section Segregational Stability of Different Constructs Carrying pMV158-Family Replicons in *L. lactis* MG1363), the small fraction of plasmid-free cells (<5% according to Equation 4 and to the estimated *L*_*ex*_, see below) that can accumulate during the growth for only five generations in the absence of antibiotic selective pressure would have a negligible contribution to the total population growth rate. Though the average growth rates of all these strains were higher in the absence than in the presence of Ery, statistically significant differences could only be found for the strains containing pLS1OM or pLS1OM-Cherry (Figure 3A,B). These results suggest that the antibiotic itself may have a slight noxious effect on the cells expressing the *ermAM* gene, although unambiguous disclosure of this effect might require performing competition experiments, which are more sensitive than monitoring the bacterial growth in pure cultures (Hernández-Arriaga et al., 2012). On the other hand, lactococcal cells containing any pLS1ROM/OM series plasmid displayed lower average growth rates in non-selective medium than the plasmid-free cells, and, except for pLS1ROM, the observed slowdown was found to be statistically significant (Figure 2E, 3A,B).

Comparison of the growth in M17G of lactococcal cells carrying different vectors (Figure 1, 2A, 3A,B) also allowed us to analyze whether transcription and/or expression of the FP genes from unrepressed promoter P_M_ impose a metabolic burden on the host. In selective medium, a slight (∼10–15%) but statistically significant decrease in the growth rate of the strain carrying pLS1OM (unrepressed transcription from P_M_) compared with the host of pLS1ROM (P_M_ transcriptional activity repressed by MalR) was inferred (Figure 3A,B). Similarly, the strain carrying pLS1OM-Cherry (unrepressed expression of *mrfp*) seemed to have a growth rate ∼10–15% slower than that containing pLS1ROM-Cherry (repressed *mrfp* expression), suggesting that unrepressed P_M_ activity may represent a certain metabolic burden for the bacterial cell (Figure 3A,B). Such a fitness cost associated to the unrepressed activity of P_M_ could not be inferred, nevertheless, when comparing the growth rates of the different lactococcal strains in non-selective medium (Figure 3A,B). On the other hand, no statistically significant differences in the rate of growth in selective or non-selective medium could be found between the hosts of pLS1ROM (repressed transcription from P_M_), pLS1ROM-GFP (repressed expression of *egfp* from P_M_) and pLS1ROM-Cherry (repressed expression of *mrfp* from P_M_), and neither between the strains carrying pLS1OM (unrepressed P_M_) and pLS1OM-Cherry (unrepressed expression of *mrfp*) (Figure 3A,B). Metabolic burden due to the unrepressed synthesis of EGFP or mCherry could neither be inferred by comparing the growth in M17M (induction conditions) of those lactococal strains harboring the inducible expression system (Figure 2B, 3C,D). Hence, the overall analysis of the growth rates of the different lactococcal strains was unable to show any fitness cost associated to the expression of the genes encoding the EGFP or mCherry FPs.

### Plasmid Copy Number of Different Constructs Carrying pMV158 or pSH71 Replicons in *L. lactis* MG1363

The number of plasmid molecules per chromosome equivalent was determined from gDNA preparations of the lactococcal strains using two different methods: (i) relative quantification of chromosomal and plasmid amplicons by qPCR, and (ii) direct densitometric quantification of the plasmid and chromosomal DNA bands on GelRed-stained agarose gels (see section Determination of Plasmid Copy Number in *L. lactis* Cells by qPCR and Agarose Gel Quantification). In this latter method, the plasmid to chromosomal DNA ratios, normalized for the plasmid size, were compared with that of pRCR12, whose qPCR-determined PCN was taken as a reference (see section Determination of Plasmid Copy Number in *L. lactis* Cells by qPCR and Agarose Gel Quantification). In some strains, the PCN was also analyzed after growing the bacterial cells for a number of generations in the absence of selection for the plasmid-encoded antibiotic resistance trait. PCNs values obtained by both methods were quite consistent for all plasmids and conditions (Table 2). This means that these two approaches can complement each other to increase the accuracy of the PCN determination. The main limitation of the qPCR is that it is not a direct method because it relies on specific DNA amplicons amplification and on an accurate determination of the efficiencies of those amplifications. Meanwhile, gel quantification is a direct method although it is less sensitive and only reliable if the DNA samples to be compared have similar fractions of the different plasmid DNA forms.

**Table 2:** Plasmid copy number of the lactococcal strains carrying the indicated plasmids.

Vector	Generations in the absence of selection for the plasmid	qPCR quantification	Gel quantification
pLS1	–	16.3 ± 2.5^A^	21.0 ± 3.7^a^
	10	N.D.	18.9 ± 2.6^a^
	50	N.D.	20.1 ± 7.9^a^
	100	N.D.	20.3 ± 4.8^a^
	150	15.1 ± 3.6	16.5 ± 1.7^a^
pMV158	–	17.7 ± 2.6^A^	23.4 ± 2.1^a^
	10	N.D.	18.6 ± 0.8^a^
	50	N.D.	18.7 ± 2.4^a^
	100	N.D.	17.6 ± 1.2^a^
	150	12.2 ± 1.0	19.8 ± 3.6^a^
pRCR12	–	113.4 ± 8.6^B^	113.4^b^
	50	N.D.	108.3 ± 5.6^b^
	100	143.4 ± 0.8^B^	110.2 ± 11.2^b^
pMV158GFP	–	42.9 ± 4.6^c^	46.0 ± 4.4^c^
	10	21.9 ± 1.2^D^	30.7 ± 3.3^d^
	50	12.3 ± 1.5^E^	12.5 ± 2.2^e^
	100	9.4 ± 0.2^E^	13.2 ± 2.7^e^
	150	7.5 ± 1.1^E, E′^	10.6 ± 0.1^e,e′^
pLS1ROM-GFP (M17G)		8.0 ± 0.3^E′^	9.2 ± 1.6^e′^
pLS1ROM-Cherry (M17G)		5.9 ± 1.2^E′^	7.2 ± 0.9^e′^
pLS1OM-Cherry (M17G)		7.1 ± 0.8^E′^	9.7 ± 1.5^e′^

*The number of plasmid molecules per chromosome equivalent was determined by qPCR or by densitometric analysis of agarose gels. In this latter method, the plasmid to chromosomal DNA ratios were compared to that of pRCR12, whose qPCR-determined PCN value was considered as a reference (red color number). The copy number values given in the table are expressed as mean ± standard deviation of three independent experiments (biological samples). All the values with the same superscript letter were not found to have statistically significant differences. N.D. not determined.*

**FIGURE 4:**
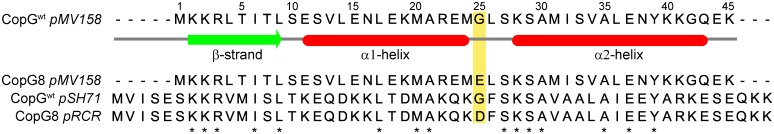
Sequence alignment of the CopG-like proteins encoded by wt and mutant replicons of the pMV158 family. CopG proteins encoded by rolling-circle replicating plasmids pSH71 and pRCR were aligned with the amino acid sequence of pMV158-encoded CopG, to which the indicated numbering and secondary structure regions correspond. The Gly residue of the turn connecting α1- and α2- helices is framed. In the CopG8 variants encoded by a copy-up plasmid mutant derived from pMV158 and by pRCR (as well as their derivatives pRCR12 and pRCR-P_M_), Glu and Asp residues substitute, respectively, the Gly of the turn.

When the host cells were grown in the presence of Tc, the pMV158GFP PCN was ∼40–45. Removal of the selective pressure for plasmid carriage resulted in a gradual decay in the average PCN until reaching ∼7–10 after 150 generations (Table 2). Since this decay was not accompanied by the appearance of plasmid-free cells (see section Segregational Stability of Different Constructs Carrying pMV158-Family Replicons in *L. lactis* MG1363), a decrease of the pMV158GFP PCN in the plasmid-carrying cells was shown to occur actually. The PCN of pMV158GFP in cells that had grown for 150 generations in the absence of antibiotic did not significantly differ from those of pLS1OM-Cherry, pLS1ROM-GFP and pLS1ROM-Cherry in lactococcal cells grown under selective pressure for the plasmid presence (Table 2). Moreover, the PCN of constructs of the pLS1ROM/pLS1OM series, which were shown to have a slightly unstable inheritance (see section Segregational Stability of Different Constructs Carrying pMV158-Family Replicons in *L. lactis* MG1363), decreased proportionally to the fraction of plasmid-containing cells (not shown) when the host strains were grown in the absence of antibiotic (Ery), indicating that the concentration of plasmid molecules was kept constant (∼6–9 plasmid copies per chromosome equivalent) in the cells that conserved the plasmid. Induction of the *egfp* or *mrfp* expression by growing the cells in the presence of maltose did not result in any significant change in the PCN of these constructs (not shown). PMV158GFP and the pLS1ROM/pLS1OM series constructs share the part of the pMV158 basic replicon involved in the controlled replication of the leading strand, although these latter plasmids lack the pMV158 *ssoU*, which is efficiently recognized in the lactococcal host for the initiation of the lagging-strand synthesis. The pLS1ROM/pLS1OM series also differs from pMV158 in the antibiotic resistance marker being Ery^R^ instead of Tc^R^ (Figure 1A). To know whether either of these genetic traits could account for the different behavior of the copy number of pMV158 and the pLS1ROM/pLS1OM series plasmids when their lactococcal hosts were grown in selective and non-selective media, we analyzed the PCN of parental plasmid pMV158 (harboring *ssoU* and *ssoA*) and its derivative pLS1 (harboring only *ssoA*), both having the *tetL* Tc^R^-determinant. In lactococcal cells grown in selective medium, the values determined for the pMV158 and pLS1 PCNs (∼20) were indistinguishable from each other, although they were found to be significantly lower and higher than those of pMV158GFP and the pLS1ROM/pLS1OM series, respectively (Table 2). Taken together, these results indicated that: (i) the presence of the *ssoU* did not modify the PCN of plasmids based on the pMV158 replicon; (ii) the decrease observed in the copy number of pMV158GFP when the host cells were grown in non-selective medium was not related to the presence of the *tetL* gene on the plasmid, and might rather result from the presence of a copy-up mutation in the plasmid pool that could represent an advantage for the host in the presence of Tc but would be counterselected in the absence of the antibiotic; and (iii) the copy number of the recombinant plasmids based on the pMV158 replicon (pMV158GFP and the pLS1ROM/pLS1OM series) is about half that of parental pMV158 and its deleted derivative pLS1, irrespective of the antibiotic-resistance determinant they harbor. Since the only heterologous DNA shared by all these recombinant plasmids is the P_M_ promoter/operator region cloned in either orientation relative to the pMV158 replicon (Figure 1A), it would appear that the sequence and/or structure of this region may cause some inhibition in the normal replication of these plasmids. To investigate whether the copy-up phenotype of pMV158GFP in the lactococcal host grown in selective medium was actually caused by a plasmidic mutation that was counterselected when the bacteria were grown in the absence of Tc, we have performed a preliminary analysis of the plasmid DNA sequence in cells grown either in the presence of the antibiotic (generation 0) or in non-selective medium for 150 generations (Supplementary Figure S1). Two different point mutations were found in the plasmidic *copG* gene encoding the replication control CopG protein that represses transcription of the essential *copG-repB* operon in the members of the pMV158 replicon family (del Solar et al., 1990, 2002). In the cells grown in the presence of Tc, positions 2 (within the initiation codon) and 49 (within the triplet encoding amino acid 17) of *copG* were found to consist of a mixture of wt (T in position 2, and C in position 49) and mutant (G in position 2, and T in position 49) nucleotides. By contrast, in the cells grown for 150 generations in the absence of Tc, position 2 remained populated by a mixture of the wt and mutant nucleotides, whereas only the wt C was observable in position 49 (Supplementary Figure S1). The ratios between the intensities of the chromatographic picks corresponding to the wt and mutant nucleotides at positions 2 and 49 of *copG* (which are inverse to each other; Supplementary Figure S1) may suggest that the total pool of pMV158GFP in cells grown in selective medium consists of a major fraction of plasmid molecules carrying the mutant initiation codon (AGG) and the wt codon 17 (CTT encoding Leu; Figure 4) along with a minor fraction of molecules containing the wt ATG initiation codon and the mutant codon 17 (TTT, encoding Phe). Mutation in position 49 of the *copG* coding sequence results in a CopG mutant having the conservative change Leu to Phe in residue 17, which is involved in intra- and inter-molecular contacts within the hydrophobic cluster of the protein (Gomis-Rüth et al., 1998). Thus, the copy-up phenotype of pMV158GFP in the lactococcal host grown in selective medium might be explained if the only CopG variant synthesized in the cells were potentially unstable and hence functionally defective. On the other hand, the wt copy-number phenotype of pMV158GFP in the host cells grown for 150 generations in non-selective medium may be accounted for by the expression of wt *copG* from a fraction of the total plasmid pool, whereas the inactive mutant gene carried by the majority of the molecules is expected to behave as a recessive allele, as it has previously been shown for other defective alleles of the repressor gene (del Solar et al., 1995).

Plasmid pRCR12, based on the pSH71 replicon, showed a PCN of ∼115 (Table 2), which was uncommonly high to correspond to a wt RC replicon from *Lactococcus*, as it should be expected to burden the host cell. We then analyzed the DNA sequence of constructs pRCR12 and pRCR-P_M_, as well as that of pRCR, from which the first two plasmids derived, and found they all harbor a single mutation in the *copG* gene of the pSH71 replicon. This mutation leads to the substitution of the conserved Gly residue located in the turn between the two α-helices of the ribbon-helix-helix CopG protein by Asp (Figure 4). An equivalent mutation (termed *copG8*) that results in the change of the CopG conserved Gly residue to Glu has been characterized in a copy-up (fivefold increase in PCN) mutant plasmid based on the pMV158 replicon (Acebo et al., 1996). The change alters the dimer-dimer interaction surface of the mutant CopG8 protein, impairing its cooperative binding to the operator DNA (del Solar et al., 2002). The mutation found in pRCR (and its derivatives pRCR12 and pRCR-P_M_) can give rise to a CopG8-like defective protein and cause the observed copy-up phenotype. No decrease in the PCN of pRCR12 was noted when the host cells were grown for 100 generations in the absence of selective pressure for the plasmid (Table 2).

### Segregational Stability of Different Constructs Carrying pMV158-Family Replicons in *L. lactis* MG1363

The stability of inheritance of pLS1ROM, pLS1ROM-GFP, pMV158GFP (based on the pMV158 replicon), and of pRCR12 (based on the pSH71 replicon) was analyzed after growing the lactococcal cells for 100 or 150 generations in the absence of selective pressure, using one or various among three different methods: (i) plate-counting estimation of the percentage of cells carrying the antibiotic resistance marker of the plasmid (the only suitable for pLS1ROM); (ii) determination of the specific fluorescence emitted by the total cell population; and (iii) direct quantification of the fraction of fluorescent cells by fluorescence microscopy.

**FIGURE 5:**
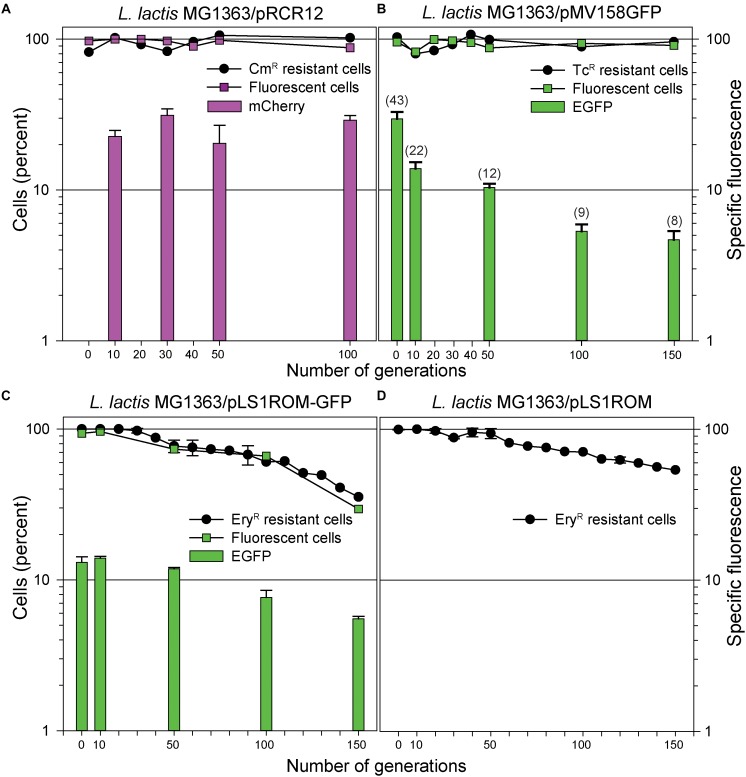
Analysis of the segregational stability of various plasmid constructs in *L. lactis* MG1363. The stability of inheritance of pRCR12 **(A)**, pMV158GFP **(B)**, pLS1ROM-GFP **(C),** and pLS1ROM **(D)** was analyzed after growing lactoccocal cells for at least 100 generations in the absence of selective pressure. An estimation of the percentage of antibiotic resistant cells at the indicated number of generations is given in the four panels. The percentage of fluorescent cells directly quantified by fluorescence microscopy is depicted at the indicated number of generations for those strains producing mCherry **(A)** or EGFP **(B,C)**. The vertical bar graph in **(A–C)** represents the specific fluorescence emitted by the total cell population at the indicated number of generations. In **(B)**, the qPCR-determined PCN of pMV158GFP at the indicated number of generations is depicted in brackets on the top of the vertical bars representing the specific fluorescence.

Plasmid pRCR12 was found to be stably inherited in the lactococcal cells, as the estimated fraction of Cm^R^ cells or fluorescent cells was kept about 100% for at least 100 generations of non-selective growth. Consistent with the values determined for the copy number of pRCR12 (Table 2), the specific fluorescence also remained quite constant over the generations, indicating that the same PCN is maintained during the bacterial growth in the absence of selective pressure for the plasmid (Figure 5A).

Segregational stability in the lactococcal host was observed as well for plasmid pMV158GFP by both estimation of the percentage of Tc^R^ cells and direct quantification of the fraction of fluorescent cells (Figure 5B). However, the specific fluorescence emitted by the cells showed a clear and gradual decrease through successive generations in non-selective medium that matched quite well the observed decline in PCN (Figure 5B and Table 2). Since the specific fluorescence reflects the average PCN in the total population, and taking into account the stable inheritance of the plasmid, the observed decrease in fluorescent emission was inferred to arise from a decrease of the number of copies of pMV158GFP in the plasmid-containing cells.

In contrast to the above plasmids, pLS1ROM-GFP was found, by both fluorescence microscopy and plate-counting, to be somewhat unstable in the lactococcal host as shown by about one third of the cells retaining the plasmid after 150 generations (Figure 5C). In fact, the decrease in specific fluorescence paralleled that of the percentage of antibiotic resistant cells. Fitting the experimental data to Equation (5) (see section Determination of Plasmid Structural and Segregational Stability in *L. lactis* of Materials and Methods) allowed us to calculate a plasmid *L*_*ex*_ of 0.0066 ± 0.0002, although a quite low R-squared value (0.93) was obtained, and an accelerated, rather than constant, loss rate was suggested from the shape of the experimental curve (Figure 5C). Plasmid pLS1ROM seemed to have an even higher stability in *L. lactis*, as reflected by the ∼55% of cells that retained the plasmid after 150 generations (Figure 5D). The *L*_*ex*_ inferred for this plasmid was 0.0044 ± 0.0002 and a slightly better fit of experimental data to Equation 5 was observed (R-square = 0.95).

The segregational instability of pLS1ROM-GFP and pLS1ROM in *L. lactis* was not related to the absence of an *sso* efficiently recognized in this bacterium (*ssoW* or *ssoU*), since plasmids pLS1 (carrying only the inefficient *ssoA*) and pMV158 (having both *ssoU* and *ssoA*) are stably inherited and no appearance of plasmid-free cells was observed during the growth of the lactococcal host for 150 generations (not shown).

### Relative Strength of Promoters P_M_ and P_X_

**FIGURE 6:**
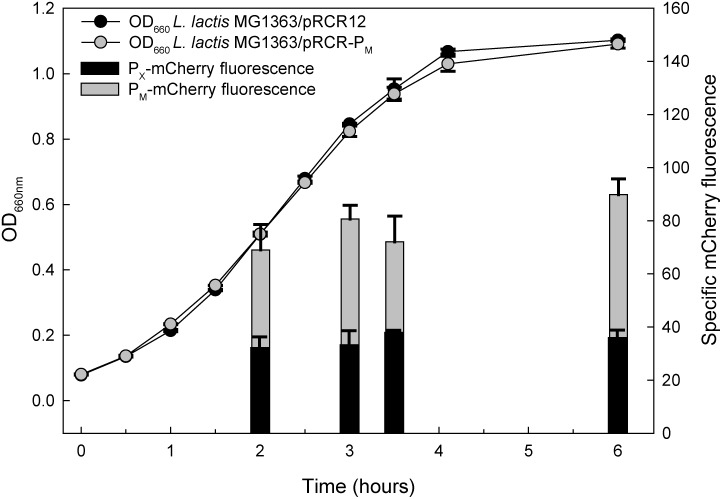
Comparative analysis of the specific fluorescence emitted by lactococcal cells carrying either pRCR12 or pRCR-P_M_ vectors. *L. lactis* cells harboring either pRCR12 or pRCR-P_M_ were grown in M17G for 6 h, without shaking, and the optical density at 660 nm was measured every 30 min using a Spectronic 20D+. The mCherry fluorescence was measured at the indicated points of the growth curve, once the cells were harvested, washed and resuspended in PBS, and then incubated 4 h at 22°C for completion of FP maturation. Vertical bars represent the specific fluorescence emitted by the lactococcal cells.

We have compared the strength of the two promoters used for directing the expression of the FP genes of the fluorescent labeling vectors studied in this work. To this end, we measured the specific red fluorescence at various points of the growth curve of lactococcal cells that contain either pRCR12 (gene *mrfp* transcribed from P_X_) or pRCR-P_M_ (*mrfp* transcribed from P_M_). In all cases, the intensity of fluorescence emitted by the cells containing pRCR-P_M_ was about twice that of the pRCR12-carrying cells (Figure 6). It is worth noting that the stronger expression of the *mrfp* gene from plasmid pRCR-P_M_ is not accompanied by any observable decrease in the host fitness, as shown by the good match between the pRCR-P_M_- and pRCR12-containing lactococcal cells in both the growth curves (Figure 6) and the growth rate constants (Figure 3A,B).

### Maturation Time of Fluorescent Proteins

The protocol commonly reported to visualize LAB fluorescently labeled with EGFP or mCherry by fluorescence microscopy, as well as to quantify their fluorescent emission, involves collecting the cells, washing and resuspending them in PBS and subsequently analyzing the fluorescence without any intentional incubation (Fernández de Palencia et al., 2000; Nieto and Espinosa, 2003; Ruiz-Masó et al., 2012; Pérez-Ramos et al., 2017). When, at the beginning of the present work, we followed this protocol with the lactococcal cells that carried plasmids encoding EGFP (i.e., pMV158GFP and pLS1ROM-GFP), reproducible results were obtained and the fluorescence of the cells resuspended in PBS increased little (if any) over time. In contrast, the specific fluorescence intensity of lactococcal cells carrying plasmids that encoded the mCherry protein (i.e., pLS1ROM-Cherry, pLS1OM-Cherry, pRCR12, or pRCR-P_M_) experimented large fluctuations from one experiment to another, and the fluorescence of the bacteria resuspended in PBS was observed to increase notably (∼10–12-fold) over time at 22°C, reaching a plateau after 3–4 h (not shown). Meanwhile, the OD of these samples remained constant for at least 8 h, and cell viability (>90%) was also conserved over this period of time (not shown). To analyze whether the increase in mCherry fluorescence emission observed in the absence of bacterial growth was due to *de novo* synthesis of the FP or to maturation of the previously synthesized protein, lactococcal cells containing pRCR-P_M_ were grown to an OD of 0.4 and resuspended in PBS lacking or containing 5 μg/mL Ery, an antibiotic that inhibits gene translation. A similar increase of the specific fluorescence over time was observed irrespective of the presence or absence of the antibiotic (not shown), demonstrating that it was due to the maturation of pre-existing mCherry. These results suggested that, although fluorescence emission of both EGFP and mCherry requires post-translational maturation of the protein chromophore (Zimmer, 2002; Miyawaki et al., 2012), that of EGFP would occur faster, so that it would have been virtually completed by the time the fluorescence was measured after collecting, washing and resuspension of the bacterial cells in PBS.

**FIGURE 7:**
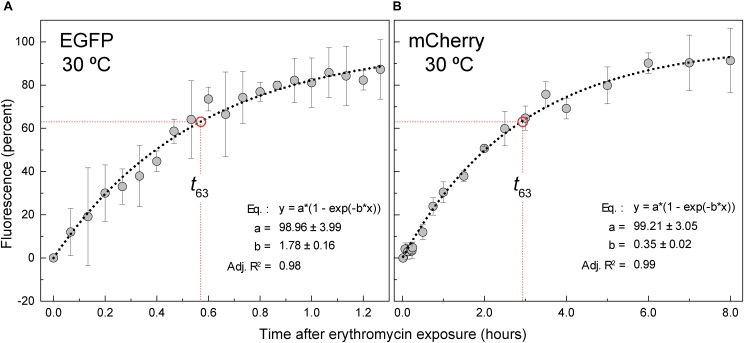
Kinetics of maturation of EGFP and mCherry. *L. lactis* cells harboring either pMV158GFP or pRCR-P_M_ were grown in AGCH_GY_ to an OD of 0.85 before addition of 5 μg/mL Ery. The fraction of mature protein was plotted against time after translational arrest. EGFP **(A)** and mCherry **(B)** maturation kinetics showed a single exponential rise to the maximum (dashed line). EGFP and mCherry fluorescence data were fitted to the equation depicted inside the graphs, which is equivalent to Equation (7). The factor *b* of the equation is equal to *1/MT*. Dashed red lines indicate the time it takes for 63% of the FP to mature (*t*_63_ EGFP = 0.56 ± 0.05 h; *t*_63_ mCherry = set operator space 2.88 ± 0.20 h). Plots display maturation kinetic curves obtained from three independent experiments (biological samples), with the symbols and vertical bars representing the mean and standard deviation, respectively.

To determine the maturation time of EGFP and mCherry in *L. lactis*, we monitored the time course of the fluorescence increase of cultures of the pMV158GFP- or pRCR-P_M_-carrying strains following inhibition of *de novo* protein synthesis by Ery (*t* = 0; Figure 7A,B). The experimental data of the specific fluorescence were fitted to Equation 7 (see section Estimation of the FP Maturation Times by Translational Arrest With Erythromycin of Material and Methods), in order to determine the *MT* (t_63_) of EGFP and mCherry in lactococcal cells grown under the specified conditions (static cultures in AGCH_GY_ at 30°C). *MTs* of 0.56 h (Figure 7A) and 2.88 h (Figure 7B) were estimated for EGFP and mCherry, respectively, showing a much slower maturation of the red FP. These results are consistent with a longer time required for maturation of mCherry compared to EGFP in *E. coli*, which has been attributed to a second slow oxidation step occurring during formation of the red FP chromophore (Hebisch et al., 2013; Balleza et al., 2018).

### Stability of EGFP and mCherry in the Lactococcal Cells

**FIGURE 8:**
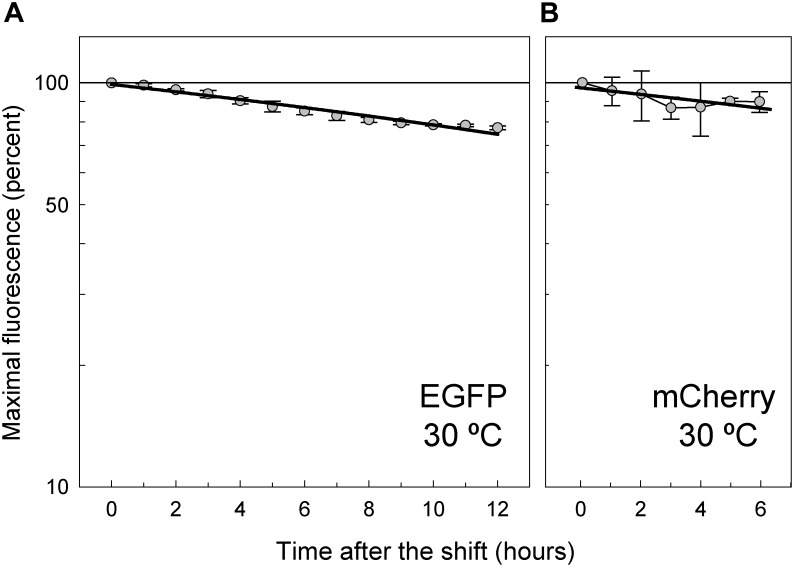
Stability of EGFP **(A)** and mCherry **(B)** in *L. lactis* MG1363. After induction of *gfp* or *mrfp* expression in the lactococcal host carrying pLS1ROM-GFP or pLS1ROM-Cherry, respectively, the bacterial cells were shifted to AGCH medium lacking any carbon source in order to stop their growth. The decay of the EGFP fluorescence intensity was analyzed for 12 h following the shift. In the case of the cultures of *L. lactis*/pLS1ROM-Cherry, and due to the slower maturation of mCherry, the fluorescence decline was analyzed during a shorter period (6 h). EGFP and mCherry fluorescence data were fitted by linear regression to Equation (9), and the *in vivo* half-life time of mature FP was calculated from the slope of the regression curve according to Equation (10). With the available data, half-lives of 30.4 ± 1.2 h and 36.7 ± 12.5 h were estimated for EGFP and mCherry, respectively. Plots display fluorescence decay curves obtained from three independent experiments (biological samples), with the symbols and vertical bars representing the mean and standard deviation, respectively.

To analyze the intracellular stability of EGFP and mCherry, as well as to determine their half-lives, we took advantage of the inducible system for expression of the corresponding FP genes carried on plasmids pLS1ROM-GFP and pLS1ROM-Cherry (Figure 1A). Cultures of *L. lactis* harboring either plasmid were induced for 8 h in the presence of maltose and subsequently shifted to medium lacking any sugar, and processed as indicated in Material and Methods (section Determination of the Half-Life of EGFP and mCherry in *L. lactis*) in order to monitor the decline of the fluorescence intensity over a period of time during which the cells stop their growth but maintain their viability (Figure 8A,B). Due to the slower maturation of mCherry, the maximal fluorescence intensity of the bacterial cells tagged with this FP was only reached 6 h after the shift, and hence the time period of the analysis was reduced to the subsequent 6 h, when the cell viability remained higher than 95% (Figure 8B). Both FPs were extremely stable in the lactococcal cells, showing half-lives longer than 24 h (30.4 ± 1.2 h and 36.7 ± 12.5 h for EGFP and mCherry, respectively), although the half-life time of mCherry could not be precisely determined due to the lower number of experimental points, which in turn results from their slower maturation, the small slope of the fluorescence decline curve, and the relatively high standard deviations (Figure 8B).

## Discussion

Despite the fact that over the last two decades plasmidic vectors have experienced a growing use among LAB as genetic tools for gene expression and characterization of regulatory regions, as well as for tracking bacterial cells, there is a general lack of information about key features (e.g., copy number, stability and fitness cost of the plasmids in the target bacterial hosts) that could condition their potential applications. Moreover, studies on the maturation kinetics and intracellular stabilities of the most common FPs are still missing in most LAB, although these parameters, which are affected by the intracellular environment and the external conditions (Hebisch et al., 2013; Balleza et al., 2018), may be critical to optimize the use of these biomarker proteins and to avoid artifacts and mistakes in the quantification of the emitted fluorescence. Using *L. lactis* as a model LAB we have investigated the main features of a series of fluorescence labeling vectors constructed, either previously or in the present work, by combining a replicon of the pMV158 family (pMV158 or pSH71) with a promoter widely recognized in Firmicutes (P_X_ or P_M_) that directs transcription of a gene encoding a FP (EGFP or mCherry). In order to achieve inducible expression of the FP gene, some of the constructs were also provided with the gene that encodes transcriptional repressor MalR, which in the absence of maltose binds with high affinity to its operator sequence in the P_M_ promoter region (Nieto et al., 2001).

High-copy number plasmids pRCR12 and pRCR-P_M_, which carry a *copG8*-like copy-up mutated version of the pSH71 replicon (Figure 4), were stably inherited in *L. lactis*, and no plasmid-free segregants were observed for at least 100 generations in non-selective medium. We have also disclosed that the pRCR promoter-probe, from which pRCR12 and pRCR-P_M_ derived, harbors the *copG8*-like mutation. Plasmid pRCR derived, in turn, from pNZ8048, wherein a DNA fragment containing the *mrfp* gene along with a preceding multiple cloning site was cloned (Mohedano et al., 2014). Although pNZ8048 has not been reported to carry a copy-up mutation, it is possible that the presence of the *copG8*-like mutation has been long overlooked. On the other hand, the number of copies of the wt pMV158 replicon per chromosome equivalent in *L. lactis* is ∼20, as it has been determined for both the parental plasmid and its derivative pLS1 (Table 2). Two kinds of constructs based on the pMV158 replicon displayed, however, altered copy-number phenotypes, namely pMV158GFP and the pLS1ROM/pLS1OM series (Table 2). Plasmid pMV158GFP (P_M_ directing expression of *egfp*) exhibited a copy-up phenotype (twice the PCN of pMV158 or pLS1) when its lactococcal host was grown in selective medium, but a low-copy number phenotype (∼8) in the bacterial cells grown for 150 generations in the absence of Tc. The observed reversion in the pMV158GFP copy-number phenotype seems to be accounted for by the expression, in the bacterial cells subjected to selective pressure, of a copy-up mutant allele of the *copG* gene, which would be counterselected and displaced by the wt replicon when selection is removed (Supplementary Figure S1). Should this be the case, a PCN of ∼8 would correspond to the copy-number phenotype of the wt pMV158 replicon in the pMV158GFP construct. Also, the PCN of plasmids of the pLS1ROM/pLS1OM series (P_M_ directing expression of *mrfp* or *egfp*) was ∼8, irrespective of the presence or absence of Ery in the culture medium of the bacterial host, indicating that this is also the copy-number phenotype of the wt replicon for these constructs, and pointing to a potential pMV158-replicon inhibitory element included in the P_M_ promoter/operator region in all these recombinant plasmids. The characterization of the PCN of plasmids carrying replicons of the pMV158 family reveals that selection of copy-up mutants in the *copG* gene is not an uncommon event in *L. lactis*. Apart from pRCR (and its derivatives pRCR12 and pRCR-P_M_) and pMV158GFP, one *copG8* mutant of pLS1 was obtained the first time we tried to introduce the latter plasmid in this bacterium (not shown). Concentration of spontaneous copy-up mutations in the pMV158 *copG* gene has previously been reported in the pneumococcal host (Acebo et al., 1996). These findings enable us to construct copy-up variants of the pLS1ROM/OM series, which can be very useful to obtain higher levels of fluorescence in the host bacterial cells.

The low PCN of the pLS1ROM/pLS1OM series in the lactococcal host could account for their unstable inheritance, as expected for plasmids that lack an active partition system and hence are randomly distributed to the daughter cells. However, the experimental loss rates of members of the pLS1ROM/pLS1OM series (apparent *L*_*ex*_ ranging from 0.004 to 0.007) were found to be much higher than the theoretical loss rates corresponding to plasmids with PCN of ∼6-9 (*L*_*th*_ ≤ 0.00024, according to Equation 6). As inferred from Equation 6, the plasmid loss rates are predicted to be very sensitive to variations in the PCN, since a decrease in a single copy leads to a fourfold increase in the *L*_*th*_. Apart from a potential overestimation in the quantification of the PCN, several factors may account for *L*_*ex*_ being higher than predicted, including fluctuations in the PCN in individual cells or reduction in the number of segregating units due to plasmid clustering and/or multimerization (although less than 15% of plasmid dimers were observed in any of the DNA samples analyzed). Moreover, if the arising plasmid-free cells have an increased fitness, they will overgrow the plasmid-bearing cells, resulting in an accelerated (instead of constant) rate of plasmid loss. In fact, the rather curved line in the graph of Figure 5C strongly suggests an accelerated plasmid loss rate, which is, in turn, indicative of a fitness cost associated with the presence of pLS1ROM-GFP. This was less evident for the stability plot corresponding to pLS1ROM (Figure 5D). Since pLS1ROM and pLS1ROM-GFP have comparable PCNs, they are expected to also show similar intrinsic rates of plasmid loss resulting from the random distribution of the copies to the daughter cells. Hence, the higher *L*_*ex*_ value inferred for pLS1ROM-GFP likely reflects a higher fitness cost of this plasmid relative to pLS1ROM. Actually, the average growth rate value determined for the host of pLS1ROM-GFP was lower than that of the pLS1ROM-containing host in either selective or non-selective medium, although the statistical analysis of the data failed to show significant differences between both strains (Figure 2E, 3A,B). A statistically significant decrease in the growth rate of the host of pLS1ROM-GFP (but not in that of pLS1ROM) with respect to the plasmid-free strain was, nevertheless, found (Figure 3A,B), pointing to a higher fitness cost of pLS1ROM-GFP. The increased metabolic burden imposed by pLS1ROM-GFP with respect to pLS1ROM might be due to its larger size or to leaky *gfp* expression. Be that as it may, the presence of pLS1ROM-GFP (and likely of pLS1ROM) seems to have a fitness cost to the lactococcal cells, which would lead to overestimation of the intrinsic rate of plasmid loss, thus explaining, at least in part, an experimental segregational stability lower than predicted. Overestimation of the plasmid loss rate also has practical implications for the potential use of pLS1ROM-GFP for labeling lactococcal cells that have to grow in non-selective medium for 10 or even 20 generations, since, in contrast to what is expected from the apparent *L*_*ex*_, only plasmid-carrying cells will be virtually present by this time.

No pMV158GFP-free segregants were observed when the lactococcal host was grown for 150 generations in the absence of selective pressure for the plasmid, although the PCN seemed to decrease gradually until a minimum value of ∼8 (which approaches what we consider the copy-number phenotype of the wt replicon in this construct, see above) is reached after 150 generations. Hence, it is possible that growth of the lactococcal host in non-selective medium for at least 100–150 more generations (depending on the fitness cost of pMV158GFP) would had been required to unambiguously detect the appearance of plasmid-free segregants.

Overall, a decrease in the host fitness of ∼15% could be associated with the carriage of any of the tested labeling vectors except pMV158GFP, which seems to impose a lower (if any) metabolic burden on the lactococcal cells (Figure 2C–E, 3A,B). The low fitness cost associated with the carriage of pMV158GFP cannot be accounted for by the presence on this plasmid of a *sso* (*ssoU*) efficiently recognized in lactococci, since no significant metabolic burden on its host is either imposed by plasmid pLS1, which lacks *ssoU* and only contains the much less efficient *ssoA* (Figure 2C, 3A,B). Highly sensitive competition assays (Hernández-Arriaga et al., 2012) might be required to accurately reveal the potential very small fitness cost due to the presence of pMV158 or its derivatives pLS1 and pMV158GFP, all of which carry the constitutively-expressed *tetL* TcR determinant (Figure 1). The small but significant reduction in host fitness caused by pRCR12 and pRCR-P_M_ may arise from the high intracellular concentration of these plasmids that carry a copy-up mutation in the *copG* gene within the pSH71 replicon (Figure 2D, 3). In the case of the low copy-number constructs belonging to the pLS1ROM/pLS1OM series, the small fitness cost associated with the plasmid carriage itself (Figure 2E) might arise from the constitutive expression of the *ermAM* Ery^R^ determinant encoding a methyltransferase that modifies the rRNA (Gupta et al., 2013).

Proper application of the FPs requires knowledge of their intracellular stability, which can vary depending on the genetic background and physiological conditions of the host bacterium. Because of its lifestyle, LAB encode numerous proline-peptidases that degrade proline-rich proteins, like the caseins (Liu et al., 2010). GFP-like FPs have proline contents similar to those of caseins (≥4%), so that we considered it interesting to determine the half-lives of the FPs used in the present study in *L. lactis*. Both EGFP and mCherry were found to be extremely stable in the lactococcal cells, with half-lives in the range of the value reported for GFPmut3 in *E. coli* (>24 h; Andersen et al., 1998).

Another important parameter to be taken into account when working with FPs is the rate of maturation of their fluorophore, which not only depends on the protein itself, but also on the external conditions (e.g., pH, temperature), as well as on the genetic and metabolic context of the bacterial cells (Hebisch et al., 2013). We have found that, in *L. lactis*, maturation of mCherry takes much longer than that of EGFP (Figure 7), which is consistent with the additional slow oxidation step required for chromophore formation in the red FP (Hebisch et al., 2013). Slower maturation of mCherry relative to EGFP has been shown to occur also in *E. coli* both at 37°C and at 32°C, and the authors (Balleza et al., 2018) warned of the potential artifacts and mistakes that can derive from diminishing the maturation effect, especially for slowly-maturating FPs, when quantification of the fluorescence emitted by the bacteria is required.

The characterization of the different modules composing the fluorescent-labeling vectors analyzed in the present work facilitates the choice of the most suitable one for a particular application.

The spectral features of the mCherry FP allow the daylight detection of red-colored colonies formed on M17 medium by lactococcal cells that express the *mrfp* gene, which facilitates the direct selection of transformants and the analysis of the plasmid segregational stability (Campelo et al., 2014). In contrast, lactococcal colonies expressing *egfp* did not show any yellow-green color under daylight, so that direct screening of plasmid-containing cfu was not feasible. However, we have shown here the suitability of the inducible *egfp* expression system carried on pLS1ROM-GFP for fluorescence microscopy direct detection of plasmid-containing cells in tests of plasmid segregational stability. Maltose-induction of the *egfp* expression in bacteria that have grown for a number of generations in non-selective medium allows preventing potential false positive associated with the high stability of EGFP, since only the cells retaining the plasmid at the moment of induction will be able to synthesize the FP. In addition, because of its shorter maturation time, EGFP provides a distribution of the fluorescence signal per cell more favorable for fluorescence microscopy than mCherry (i.e., a greater intensity and a lower fraction of cells below the fluorescence detection threshold) (Balleza et al., 2018). Regarding the use of fluorescently labeled bacteria in interactomic studies, a source of concern is the autofluorescence of the eukaryotic cells and tissues, which may hinder the detection of the FP specific fluorescence. Autofluorescence is mainly caused by riboflavin, flavin coenzymes and flavoproteins, whose spectroscopic characteristics (excitation at 450–490 nm and emission at 520–560 nm) are similar to those of EGFP although differ from those of mCherry (Billinton and Knight, 2001). In fact, because of their small spectral overlap, the FPs encoded by the plasmid constructions analyzed in this work (namely, EGFP and mCherry) are most suitable for differential labeling and simultaneous tracking of two distinct bacterial strains (Nancharaiah et al., 2003; Barbier and Damron, 2016). It is worth noting that the intensity of fluorescence emitted by fluorescently tagged bacteria depends on both the dosage of the gene encoding the FP (i.e., the PCN) and the strength of the promoter directing transcription of this gene. In this sense, the labeling vectors investigated here provide a range of PCN (∼8–115; Table 2) along with two alternative levels of expression of the FP genes, depending on whether promoter P_X_ or promoter P_M_ (the latter being twice as strong as the former in *L. lactis*; Figure 6) directs their transcription. Although the low PCN (∼8) of some of the constructs analyzed here likely entails their slightly unstable inheritance (Figure 5), an exploitable advantage of using segregationally-unstable plasmid vectors as tools for bacterial tagging or gene expression is that they can be easily removed once the task they were designed for has been accomplished.

Based on the substantial effect that the maturation process causes on the mCherry fluorescence intensity (see section Maturation Time of Fluorescent Proteins and Figure 7), we conclude that the study of the kinetics of fluorophore maturation should deserve more thorough consideration than it has previously been given, at least in LAB. Knowing the maturation time of the FP to be used for tagging a given bacterial strain allows maximizing the fluorescence signal, and is crucial to avoid artifacts and inaccuracies, especially in those experiments that involve quantitation of the intensity of fluorescence emitted by a slow-maturating FP (Balleza et al., 2018). In fact, an accurate comparative quantification of *mrfp* expression by monitoring mCherry fluorescence would require either (i) to stop bacterial growth (and concomitant protein synthesis) and bring virtually all the preexisting FP to maturation completion, or (ii) to measure the fluorescence signal in growing bacteria and correct with a factor that quantifies the interplay between the FP maturation time and the protein dilution due to cell growth (Balleza et al., 2018). In both cases, estimation of the FP maturation time is required.

All plasmid vectors analyzed in this work contain promiscuous replicons of the pMV158 family, which have shown to replicate in many species belonging to the Firmicutes, Actinobacteria and Proteobacteria phyla, and hence they were designed to be potentially used across a wide range of bacteria, including LAB (Table 1). It should be highlighted, however, that the main features displayed by each of these vectors (namely PCN, segregational stability, host fitness cost, relative strength of the promoter directing transcription of the FP gene, intracellular stability and maturation time of the FP) are species- and even strain-specific, since they can be affected by the presence of other plasmids, and by the metabolic context of the bacterial cells (Hebisch et al., 2013). Thus, the results obtained here for *L. lactis* MG1363 cannot be directly extrapolated to other LAB, and characterization of the different labeling vectors in the particular bacteria to be tagged would be required.

## Conclusion

Labeling of LAB with FPs can greatly facilitate tracking of the bacterial cells in several *in vitro* and *in vivo* studies including biofilm formation, competition, survival under stress conditions, and interaction with eukaryotic cells. The present study compiles valuable information highlighting the importance of a detailed characterization of the labeling vectors, as well as of the maturation and stability properties of the FP they encode, in order to optimize their use for a particular application in a given host, and avoid artifacts in the quantification of the emitted fluorescence. This knowledge could be critical for making the best choice among the available vectors aimed at the fluorescent labeling of LAB for a variety of studies.

## Data Availability

The datasets generated for this study are available on request to the corresponding author.

## Author Contributions

JG-N, DG-M, and JR-M contributed to the acquisition and analysis of the data, prepared the figures, and wrote the Materials and Methods section. JB and GS contributed to the conception and design of the study. GS contributed to the statistical analysis and wrote most of the manuscript. All authors contributed to the manuscript revision, read, and approved the submitted version.

## Conflict of Interest Statement

The authors declare that the research was conducted in the absence of any commercial or financial relationships that could be construed as a potential conflict of interest.
