# Supplementary material for: Combining Modules for Versatile and Optimal Labeling of Lactic Acid Bacteria: Two pMV158-Family Promiscuous Replicons, a Pneumococcal System for Constitutive or Inducible Gene Expression, and Two Fluorescent Proteins
Source: Front Microbiol. 2019 Jun 26;10:1431. doi: 10.3389/fmicb.2019.01431 (PMC6607859; doi:10.3389/fmicb.2019.01431)
Supplement: Supplementary file 1 [file Data_Sheet_1.docx]

Supplementary Material

**Combining Modules for Versatile and Optimal Labeling of Lactic Acid Bacteria: Two pMV158-Family Promiscuous Replicons, A Pneumococcal System for Constitutive or Inducible Gene Expression, and Two Fluorescent Proteins**

Javier Nicolás Garay-Novillo^1,2†^, Diego García-Morena^1†^, José Ángel Ruiz-Masó^1†^, José Luis Barra^2^, Gloria del Solar^1,*^.

^1^Microbial and Plant Biotechnology Department. Centro de Investigaciones Biológicas, Consejo Superior de Investigaciones Científicas, Madrid, 28040, Spain.

^2^Centro de Investigaciones en Química Biológica de Córdoba (CIQUIBIC-CONICET). Haya de la Torre s/n. Córdoba X5000HUA, Argentina.

*** Correspondence:**Gloria del Solar
[gdelsolar@cib.csic.es](mailto:gdelsolar@cib.csic.es)

| Sequence of primers used for real-time qPCR | | | | | |
| --- | --- | --- | --- | --- | --- |
| Target | Accession No. | Sequence (5’-3’)^a^ | Length (nt) | Primer position | Product size (bp) |
| *pcrA* | NC_009004.1 | F: TCAGGTGGTTTTGCAAGTGGA | 21 | 1410756-1410735 | 142 |
|  |  | R: TACGATGAATGGCGGTGTCC | 20 | 1410611-1410630 |  |
| *tetL* | NC_010096.1 | F: TGCGAGTACAAACTGGGTGA | 20 | 1879-1898 | 146 |
|  |  | R: ACCCAATTACCGACCCGAAA | 20 | 2024-2005 |  |
| *repB* | JN381945.1 | F: AGACCGAACTTTTCAATGTGGT | 22 | 1268-1289 | 146 |
|  |  | R: AGTTTTCCCTGCAATAACTTCGT | 23 | 1413-1391 |  |
| *mrfp* | pRCR partial sequence | F: TGAACTTTGAAGACGGTGGC | 20 | - | 148 |
|  |  | R: CTCGCTTCCCAACCCATAGT | 20 | - |  |
| Sequence of primers used for iPCR | | | | | |
| Name | Accession No. | Sequence (5’-3’)^a^ | Length (nt) | Primer position | Product size (bp) |
| DelmalR1 | JN381945.1 | F: GAGACTGGGCAAAAAGTCGTTA | 22 | 2973-2794 | 5415 |
| DelmalR2 | JN381945.1 | R: ATTCTTTTCTTCATCATCGGTCA | 23 | 1582-1560 |  |
| Sequence of primers used for new plasmid constructions | | | | | |
| Fmcheclon | pRCR12 partial sequence | F: GCAACCGTTTTCTATTTGTGC | 21 | - | 857 |
| Rmcheclon | pRCR partial sequence | R: GAGCTCATTTATATAATTCGT | 21 | - |  |
| SecMCSgfp | JN381945.1 | F: GATATAGGCGCCAGCAACC | 19 | 5499-5517 | 385 |
| M2 | JN381945.1 | R: TGTGGGAAATTTAGGCGCAC | 20 | 5883-5864 |  |
| Sequence of primers used for pMV158 replicon automated DNA sequencing | | | | | |
| Name | Accession No. | Sequence (5’-3’)^a^ | Length (nt) | Primer position | Product size (bp) |
| dso1 | NC_010096.1 | F: AGGGAGATGTTGTGGGGGAT | 20 | 388-407 | 1193 |
| crep2 |  | R: TCTTTTCTTCATCATCGGTC | 20 | 1580-1561 |  |

^a^ F and R indicate forward and reverse primers, respectively.

**Supplementary Table S1. Oligonucleotides used in this study**


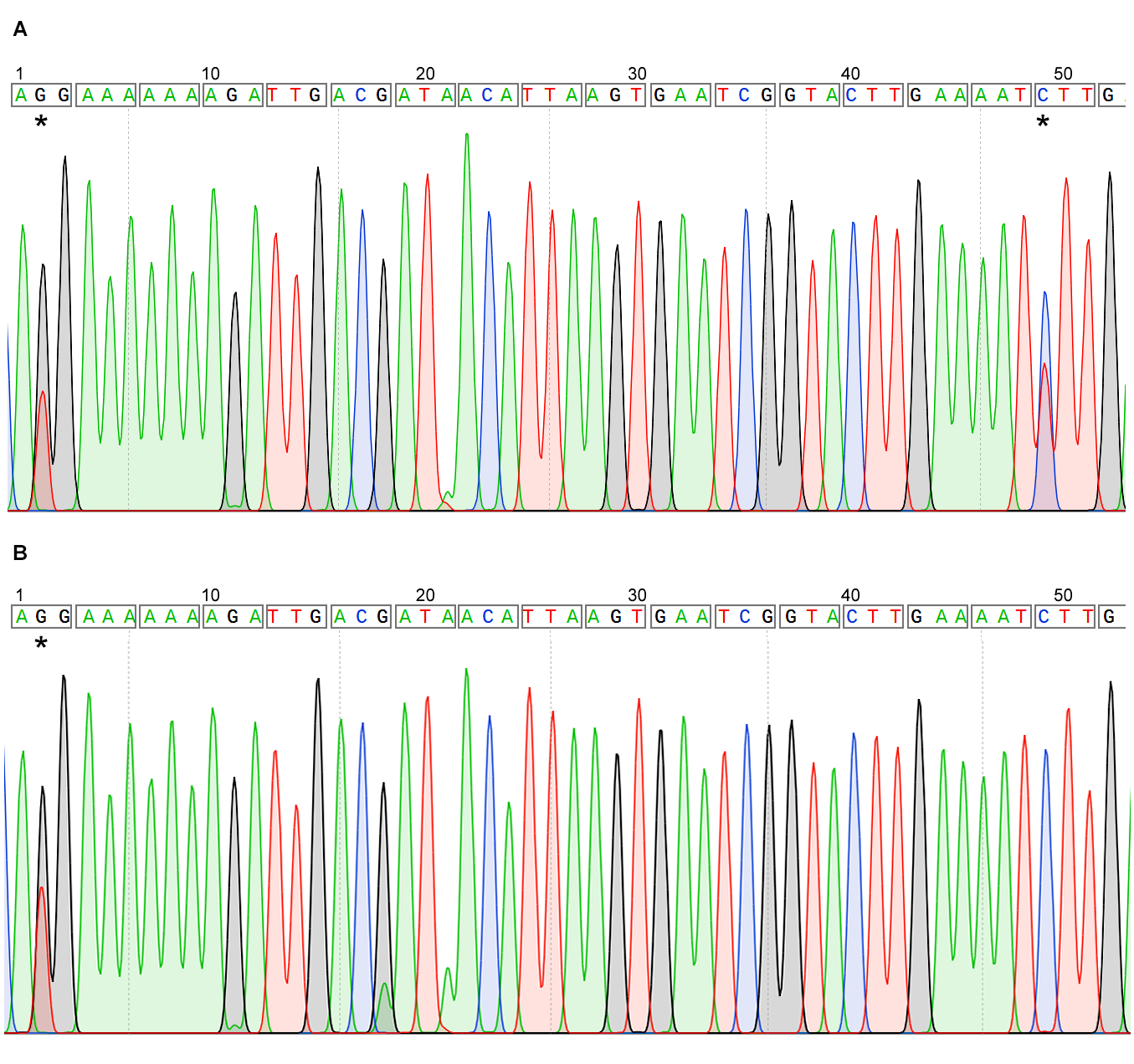


**Figure S1. Analysis of the sequence of the *copG* gene of pMV158GFP.** Chromatograms generated from DNA sequencing reactions by using dso1 and crep2 primers (Table S1) and pMV158GFP plasmid DNA template obtained from cells grown in the presence of Tc (generation 0) (**A**) or in non-selective medium for 150 generations (**B**). The black asterisks indicate positions in *copG* gene sequence with altered nucleotides. In panel **A**, positions 2 (within the initiation codon) and 49 (within the triplet encoding amino acid 17) of *copG* were found to consist of a mixture of wt (T in position 2, and C in position 49) and mutant (G in position 2, and T in position 49) nucleotides. In panel **B**, position 2 remained as a mixture of peaks corresponding to the wt and mutant sequence, whereas only the wt C signal was observable in position 49.
